# Expression Regulation Mechanisms of Sea Urchin (*Strongylocentrotus intermedius*) Under the High Temperature: New Evidence for the miRNA-mRNA Interaction Involvement

**DOI:** 10.3389/fgene.2022.876308

**Published:** 2022-06-29

**Authors:** Lingshu Han, Zijiao Quan, Yanglei Wu, Pengfei Hao, Wenpei Wang, Yuanxin Li, Xianglei Zhang, Peng Liu, Chuang Gao, Heng Wang, Luo Wang, Weijie Zhang, Donghong Yin, Yaqing Chang, Jun Ding

**Affiliations:** ^1^ Ningbo University, Ningbo, China; ^2^ Key Laboratory of Mariculture & Stock Enhancement in North China’s Sea, Ministry of Agriculture and Rural Affairs, Dalian Ocean University, Dalian, China

**Keywords:** strongylocentutros intermedius, high temperature stress, Transcriptome, miRNA-mRNA interaction analysis, expression regulation

## Abstract

In the context of global warming and continuous high temperatures in the northern part of China during summer, the mortality rate of our main breeding species, *Strongylocentrotus intermedius,* reached 80% in 2020. How sea urchins respond to high temperatures is of great concern to academia and industry. In this study, we examined the antioxidant enzyme activities of different color tube-footed sea urchins under heat stress and compared their transcriptome and microRNA (miRNA) profiles using RNA-Seq. The results showed that the antioxidant enzyme activities of sea urchins were altered by thermal stress, and the changes in peroxidase activities of red tube-footed sea urchins were particularly significant. Investigations revealed that 1,079 differentially expressed genes (DEGs), 11 DE miRNAs, and 104 “DE miRNA-DEG” pairs in total were detected in sea urchins under high temperature stress. Several mRNA and miRNAs were significantly changed (e.g. *HSP70*, *DnaJ11*, *HYAL, CALR,* miR-184-p5, miR-92a, miR-92c, and miR-124-p5), suggesting these genes and miRNAs exerted important functions in response to high temperature. At the transcriptional level, red tube-footed sea urchins were found to be more sensitive to high temperature and could respond to high temperature rapidly. DE miRNA-mRNA network showed that miR-92b-3p and PC-5p-7420 were the most corresponding miRNAs. Five mRNAs (*DnaJ11, SAR1B, CALR, HYOU1, TUBA*) may be potential markers of sea urchin response to high temperature. Possible interaction between miRNA-mRNA could be linked to protein folding in the endoplasmic reticulum, Phagosomes, and calcium transport. This study provides a theoretical basis for the molecular mechanism of sea urchin heat tolerance and information that will aid in the selection and breeding of sea urchins with high temperature tolerance.

## Introduction

Global warming is profoundly affecting the nearshore and marine environments, resulting in environmental changes such as increased seawater surface temperature, acidification and hypoxia, which seriously threaten the population reproduction of marine organisms and the sustainable development of aquaculture (Blencowe, 2006; Sanford and Kelly, 2011; Somero, 2012). Temperature is the most fundamental environmental factor that is extensively affecting the survival of marine life (Harvell et al., 2002). Studies have shown that rising temperature can cause significant changes in the metabolism of marine organisms, which in turn cause changes in their growth, reproduction, behavior, and distribution (García-Echauri et al., 2020; Gouda and Agatsuma, 2020; Strøm et al., 2020). Negative impacts on marine organisms are frequently reported when the environmental temperature increases beyond their thermal optimum (Chan et al., 2019). In recent years, abnormally high temperatures along the coast have caused large-scale deaths of marine organisms, which have become the focus of fishery resource protection and the aquaculture industry (Yasuhara and Danovaro, 2016; Brown et al., 2019).

The sea urchin is a representative species of echinoderms and a model organism for embryonic development, and it is also an important marine fishery resource worldwide (Paredes, 2016). *Strongylocentrotus intermedius* is the major species of sea urchin cultured in China. However, it is a cold-water species and is sensitive to changes in temperature. The optimal water temperature for *S. intermedius* in its natural habitat is <20°C, but mortality can occur when the water temperature rises above 25°C (Chang et al., 2016). In recent years, water temperatures in China’s northern Yellow and Bohai Seas have frequently exceeded 25°C in summer due to global ocean warming (Zeng et al., 2006). A death rate as high as 80% of *S. intermedius* cultured in the sea areas adjacent to Shandong and Liaoning resulted from high temperatures in northern China during the summers of 2017 and 2020 (Chang, 2004). The tube foot of the sea urchin is the major tissue that comes in contact with the external environment as it participates in functions such as attachment, movement, sensing, and feeding (Siikavuopio et al., 2012). First discovered in 2010 that the pigment cell composition of *S. intermedius* tube feet may be split into very distinct red and white groups (Zhang et al., 2010). Thus far, temperature studies on red and white tube-footed sea urchin populations have mostly focused on growth and physiological and biochemical indicators (Zhao et al., 2011; Ding, 2014; Ding et al., 2015; Ding et al., 2017), whereas the molecular mechanisms underlying the differences in thermal resistance between red and white tube-footed sea urchins have not been illuminated.

In recent years, the RNA-Seq method has been widely adopted to investigate the gene response of aquatic animals to temperature stress (Samanta et al., 2006; Jiang et al., 2014; Wang et al., 2019). Studies over the past 5 years have provided important information on the gene expressions of echinoderms that were exposed to temperature stress, and the RNA-Seq results have been used to identify a number of candidate genes associated with the immune response, apoptosis pathway, and metabolic pathways, such as those of the heat shock proteins (*HSPs*), calmodulin-1 (*Cam1*), cyclophilins (*Cyps*) (Gonzalez et al., 2017; Li et al., 2019; Shi et al., 2020; Li et al., 2021). MicroRNAs (miRNAs) are thecriticaly regulators of post-transcriptional gene expression, and they perform important roles in various biological functions such as metabolism, immunity and development (Bartel, 2004; Nigam, 2014; Stepicheva and Song, 2015; Pan et al., 2021). Previous studies have recognized the critical role played by miRNAs during high temperature stress in aquatic animals (Li and Xu, 2018; Zheng et al., 2018; Ma et al., 2019). Studying gene and miRNA expression dynamics can illuminate the regulatory mechanisms of aquatic animals in response to environmental stresses. By combining RNA and miRNA analyses, a recent study identified a genetic advantage in the strong vigor group of Chinese mitten crab (*Eriocheir sinensis*) in terms of tolerance to nitrite stress. Meanwhile, several key genes involved in signal transduction, oxidative phosphorylation, and energy metabolism (such as *HSP70*, *MTND2*, *ACT7*, *HSPG2*, and *Cam1*) were detected. In addition, several miRNAs mediating calmodulin-related genes (miR-31, miR-215, and miR-142-x) were identified, which suggested that the calcium signaling pathway is involved in the response to nitrite stress in crabs (Fu et al., 2021). Qiang et al. (2017) constructed RNA and miRNA expression profiles of genetically improved tilapia farmed under high temperature stress and identified 28 differentially expressed (DE) miRNAs and 744 DE mRNAs as well as 64 negative miRNA-mRNA interactions. However, there has been no detailed investigation of miRNAs in response to heat stress in echinoderms, and currently, there is limited information on the effects of and the regulatory pathways involved in heat stress in *S. intermedius*.

This study is the first report of mRNA-miRNA interactions in *S. intermedius* under high temperature stress. We analyzed the physiological effects of heat shock on red and white tube-footed *S. intermedius*, detected and compared the dynamic changes of mRNAs and miRNAs in red and white tube-footed *S. intermedius* populations under high temperature stress, and provided clues to mRNA-miRNA interactions. These data help to understand the regulatory mechanism of *S. intermedius* on high temperature stress, provide information for screening molecular markers related to heat resistance and cultivating resistant sea urchins, and lay a foundation for healthy sea urchin farming.

## Methods

### Experimental Animals and Treatment

**FIGURE 1:**
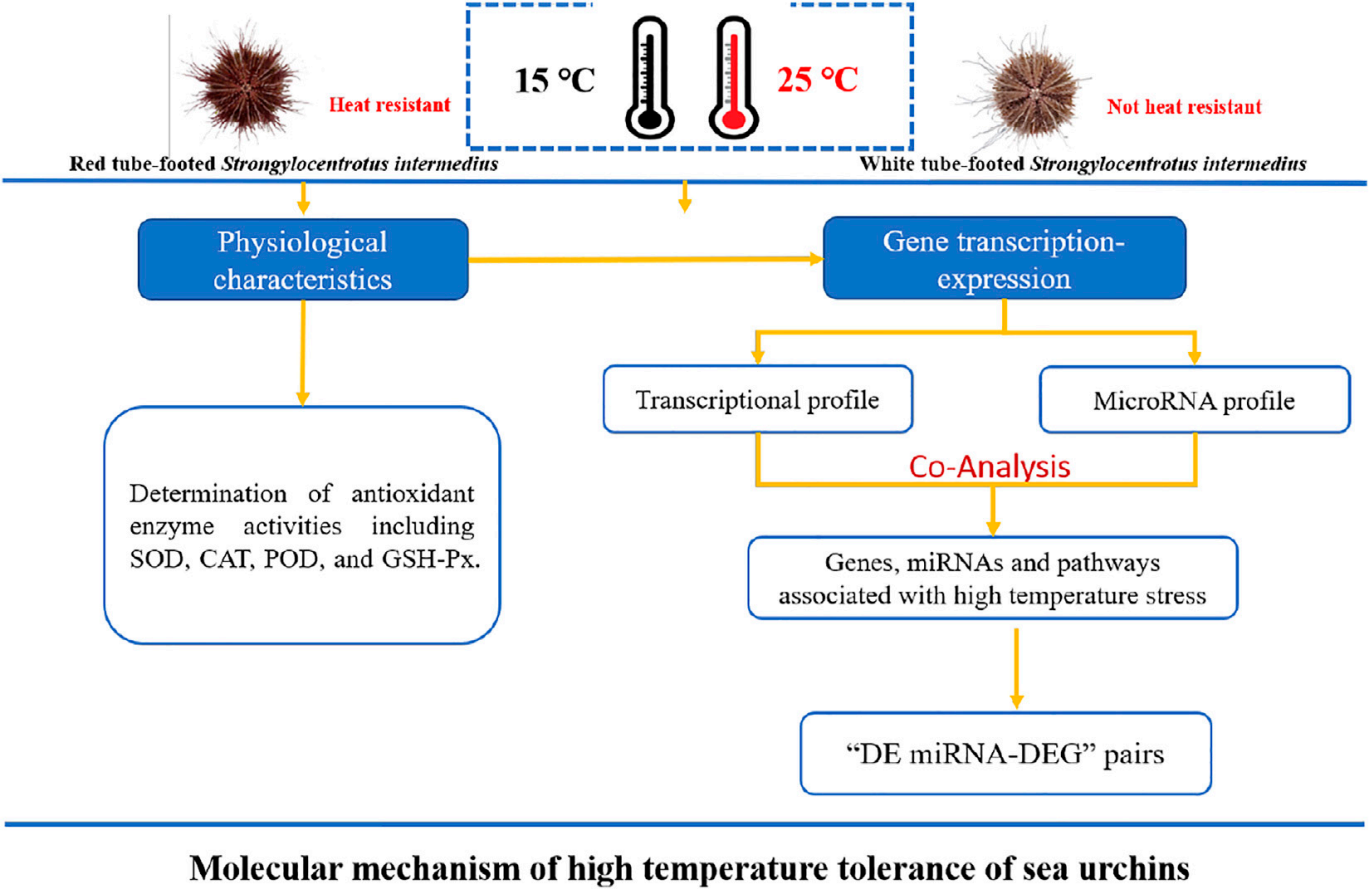
Experimental design diagram: Molecular mechanism of high temperature tolerance of sea urchins.

1.5-year-old healthy sea urchins were purchased from Dalian Lushun Haibao Fishery Company (121°23′ E, 38°17′ N) and immediately transferred to the Key Laboratory of Mariculture & Stock Enhancement in North China’s Sea, Ministry of Agriculture and Rural Affairs, P. R. China. Fifty healthy and active sea urchins with contrasting red and white tube feet of similar size (shell height 24.53 ± 2.09 mm, shell diameter 43.82 ± 2.99 mm, weight 25.73 ± 3.90 g) were selected for the experiment. Before conducting the experiments, the sea urchins were acclimatized in two 0.5 m³ temperature-independent recirculating pools (water temperature maintained at 15 ± 0.5°C, salinity 31 ± 0.5‰, pH 8.2 ± 0.2) for 1 week. During the acclimation period, the sea urchins were fed fresh kelp daily, and the water was changed by 1/2, continuously aerated. After 48 h of fasting, the red and white tube-footed sea urchin population was randomly divided into 4 groups, namely, red tube-footed sea urchin control group (NR), white tube-footed sea urchin control group (NW), red tube-footed sea urchin high temperature group (HR), and white tube-footed sea urchin high temperature group (HW). The experimental design is shown in Figure 1.

The control groups were the same as the acclimation groups, 15°C, and the experimental groups were 25°C (warmed at a rate of 2°C per hour), which was the lethal temperature for intermediate spherical sea urchins in our previous study. After reaching the temperature and maintaining it for 7 d, all sea urchins in each group were counted and individually weighed. Then, coelomic fluid of 15 randomly selected sea urchins from each group was extracted using 1 ml sterilized syringes. The upper fluid was obtained from the coelomic fluid by centrifugation (3,000 r/min, 4°C, 10 min) for determining the activities of antioxidant enzymes. The lower precipitates were used for RNA extraction. All samples were frozen in liquid nitrogen and stored at −80°C.

### Antioxidant Enzyme Activity Assays

Three sea urchin coelomic fluids were randomly selected from each group to detect antioxidant enzyme activities, including superoxide dismutase (SOD), catalase (CAT), peroxidase (POD) and glutathione peroxidase (GSH-Px), according to the instructions in the kit. All antioxidant enzyme test kits were purchased from the Nanjing Jiancheng Biological Engineering Institute (Nanjing, China).

### RNA Library Construction and Sequencing

Three coelomocyte samples were selected from every group of sea urchins. The extraction, quality testing, and purification of total RNA were performed according to Wang’s method (Wang et al., 2019). High purity 1.5 µl of total RNA from each sample was interrupted, then was reverse-transcribed to construct cDNA libraries according to the instructions of the Illumina TruSeq RNA Sample Preparation Kit (Illumina, United States). Finally, paired-end sequencing was performed on an Illumina Novaseq™ 6,000 (LC Sciences, United States). 12 mRNA libraries of sea urchins (three replicates of NR, NW, HR and HW) were built.

### Mapping and Transcriptome Assembly

To explore the changes in gene expression profiles of different colored tube-footed sea urchin populations under high-temperature stress, we obtained high-quality clean data by removing missing bases and low-quality bases (base quality ≤10) (Martin, 2011), mapped it to the reference genome (not yet published) using hisat2, and assembled the transcriptomes of red and white tube-footed sea urchin coelomocyte as well as their coelomocyte under high-temperature stress using Trinity 2.4.0 (Grabherr et al., 2011). After the final transcriptome was generated, StringTie and ballgown (http://www.bioconductor.org/packages/release/bioc/html/ballgown.html) were used to estimate the expression levels of all transcripts and perform expression level for mRNAs by calculating FPKM (FPKM = [total_exon_fragments/mapped_reads (millions) × exon_length (kB)], command line: ∼stringtie -e -B -p 4 -G merged. gtf -o samples. gtf samples. bam).

### Identification of Differentially Expressed Genes

Identification of DEGs was performed with edgeR software. The false discovery rate (FDR) was obtained by correcting the *p*-value using the Benjamini Hochberg’s method. However, since very few genes were screened with FDR in this study, in order to expand the number of differential genes and find more differentially expressed genes, *p* ≤ 0.05 and |log_2_FoldChange| >1 were considered as the threshold to determine DEGs. All the DEGs were annotated according to GO (GOseq 1.10.0 method) and KEGG (KOBAS 2.0.12 method) (Mao et al., 2005; Young et al., 2010). *p* < 0.05 was set as a filtering threshold to detect significantly enriched GO entries and KEGG pathways associated with high temperature stress.

### Small RNA Library Construction, Sequencing, and Annotation

The small RNA (sRNA) libraries were constructed with TruSeq small RNA sample prep kits (Illumina, San Diego, United States) and sequenced using Illumina HiSeq2500 (LC Sciences, Houston, Texas, United States). The obtained sequences with 18–25 nt were used to perform BLAST analysis in miR Base 22.0 database (http://microrna.sanger.ac.uk) to identify miRNAs and examine their variations.

### Identification, Target Gene Prediction, and Functional Analysis of Differentially Expressed miRNAs

To identify DE miRNAs between treatment group (HR, HW) and control group (NR, NW), the expression levels of miRNAs in the whole library were normalized, and then DE miRNAs were screened using Student’s T-test. *p* < 0.05 was considered significant. Target genes of miRNAs were predicated by using Target Finder (http://www.targetfinder.org) and were subjected to the enrichment analysis of functions and pathways by GO and KEGG database (*p*-value ≤ 0.05).

### Interaction Analysis of miRNA-mRNA

All possible positively and negatively correlated miRNA-mRNA pairs were predicted using ACGT101-CORR1.1. Based on the comprehensive analysis of DEG and DE miRNAs, we screened the negatively associated DE miRNA-mRNA pairs and constructed interaction networks using Cytoscape software (http://www.cytoscape.org/).

### Validation of DE miRNAs and mRNAs Using qRT-PCR

To validate the results of RNA-seq and miRNA-seq, 10 DEGs and 10 DE miRNAs were randomly selected for real-time fuorescence quantitative PCR (qRT-PCR). Total RNA extraction and reverse transcription were performed by referring to Han et al. (2019). 18s rRNA and U6 RNA were used as internal reference gene for qRT-PCR, which was conducted with the LightCycler96 Real-time System (Roch, Switzerland) and followed by the manufacturer’s instructions of the FastStart Essential DNA Green Master (Roch, Switzerland). The PCR primers were designed and synthesized by Shanghai Sangon Biotech and the primer sequences are shown in Supplementary Table S1. The qRT-PCR was performed in a 20 μl reaction sample containing 2 μl of cDNA (50 ng/μl), 10 μl of FastStart Essential DNA Green Master, 6 μl of ddH_2_O water, and 1 μl (10 μM) of each primer. The reaction conditions were followed by 40 cycles of 95°C for 30 s, 95°C for 5 s, 60°C for 32 s, 95°C for 15 s, 60°C for 60 s, 95°C for 15 s and 60°C for 15 s. Compared with the control gene, fold-change of expression levels was determined using 2^−ΔΔCT^ method (Livak and Schmittgen, 2001), both in miRNA and mRNA PCR amplification. Statistical analysis was performed using SPSS software version 19.0 (IBM, Armonk, NY, United States). The data were expressed as the mean ± standard error (SEM) (n = 3). In the results of gene expressions, significant differences (*p* < 0.05) for each variable were first detected using the one-way ANOVA test between different groups, followed by Tukey’s HSD test.

## Results

### Activities of Antioxidant Enzymes

**FIGURE 2:**
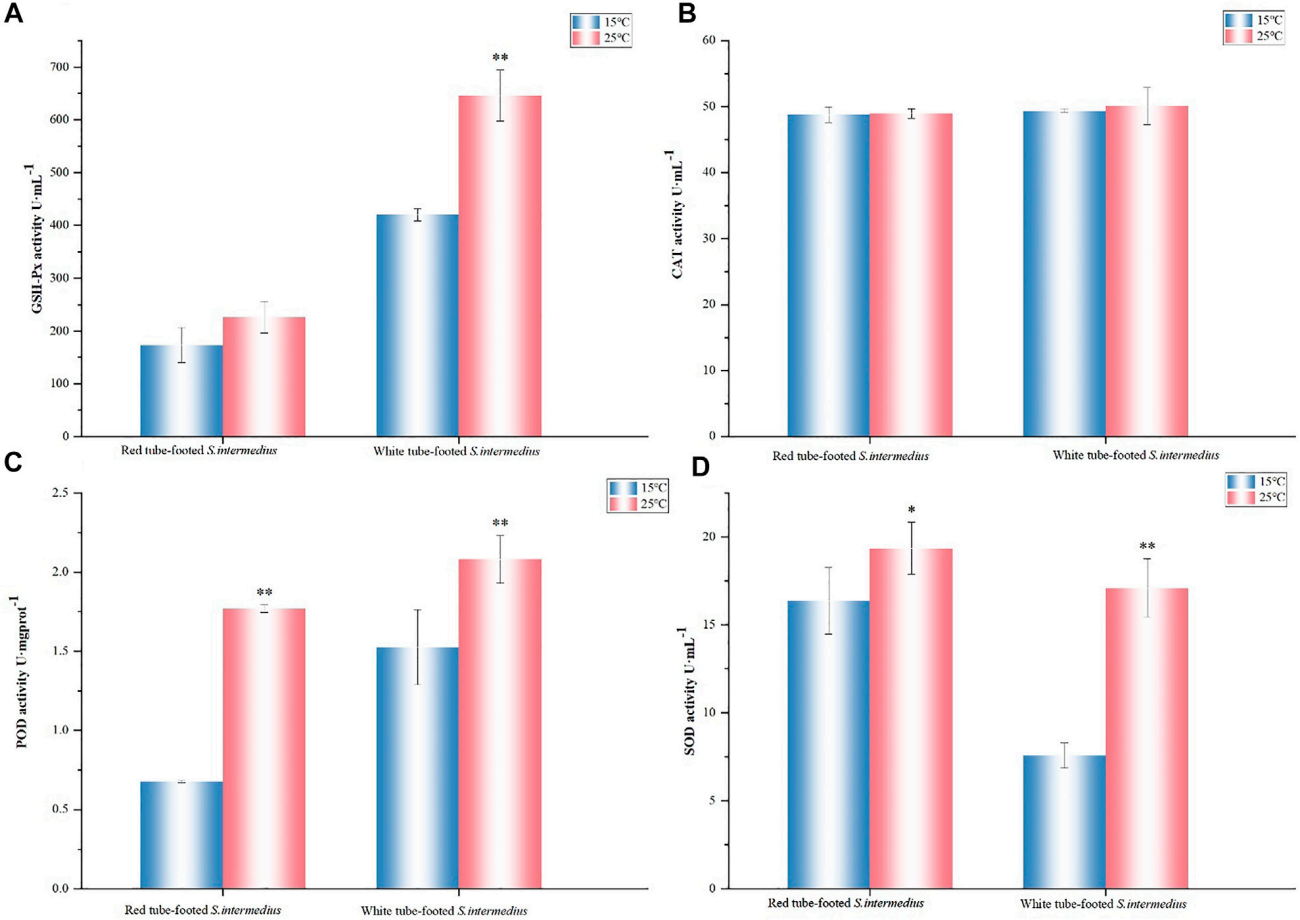
Antioxidant enzyme activity determination. **(A)** GSH-Px activity, **(B)** CAT activity, **(C)** POD activity, **(D)** SOD activity. The results are expressed as mean ± SEM (n = 3). Statistical analyses of qRT-PCR data were analyzed with independent t tests. * Significant differences at *p* < 0.05 vs control (25 vs. 15°C), ** Highly significant differences at *p* < 0.01 vs control (25 vs. 15°C).

Under high temperature stress, catalase (CAT) expression in the coelomic fluid of *S. intermedius* showed almost no change, whereas superoxide dismutase (SOD) and peroxidase (POD) expressions both increased significantly (*p* < 0.05), with greater changes in SOD in the white tube-footed *S. intermedius* and greater changes in POD in the red tube-footed *S. intermedius*. There was no significant change in glutathione peroxidase (GSH-PX) activity in the coelomic fluid of red tube-footed *S. intermedius* (*p* > 0.05), while the GSH-PX activity in the coelomic fluid of white tube-footed *S. intermedius* showed an increasing trend (Figure 2).

### Transcriptional Profiles of Red and White Tube-Footed Sea Urchin Responses to High Temperature

Twelve mRNA libraries were built and sequenced, six from 15°C control groups (NR-1, NR-2, NR-3, NW-1, NW-2, NW-3) and six from 25°C treatment groups (HR-1, HR-2, HR-3, HW-1, HW-2, HW-3). The raw reads ranged in number from 38,812,224 to 56,834,206 (Bioproject ID: PRJNA783358). After quality control, approximately 80.26–97.57% of raw reads remained as clean reads (Supplementary Table S2). The mapping results showed that 45.79–70.20% of clean reads were mapped to the reference genome sequence (Supplementary Table S3), of which, on average, 80.20, 5.25, and 14.55% of the reads were mapped to the exon, intron and intergenic regions, respectively (Supplementary Table S4).

### Identification and Functional Annotation of the Differentially Expressed Genes

On the basis of the sequencing data of red and white tube-footed *S. intermedius* in the normal group at 15°C (NR, NW) and under high temperature stress at 25°C (HR, HW), gene expression comparisons between control and treatment groups were undertaken.

**FIGURE 3:**
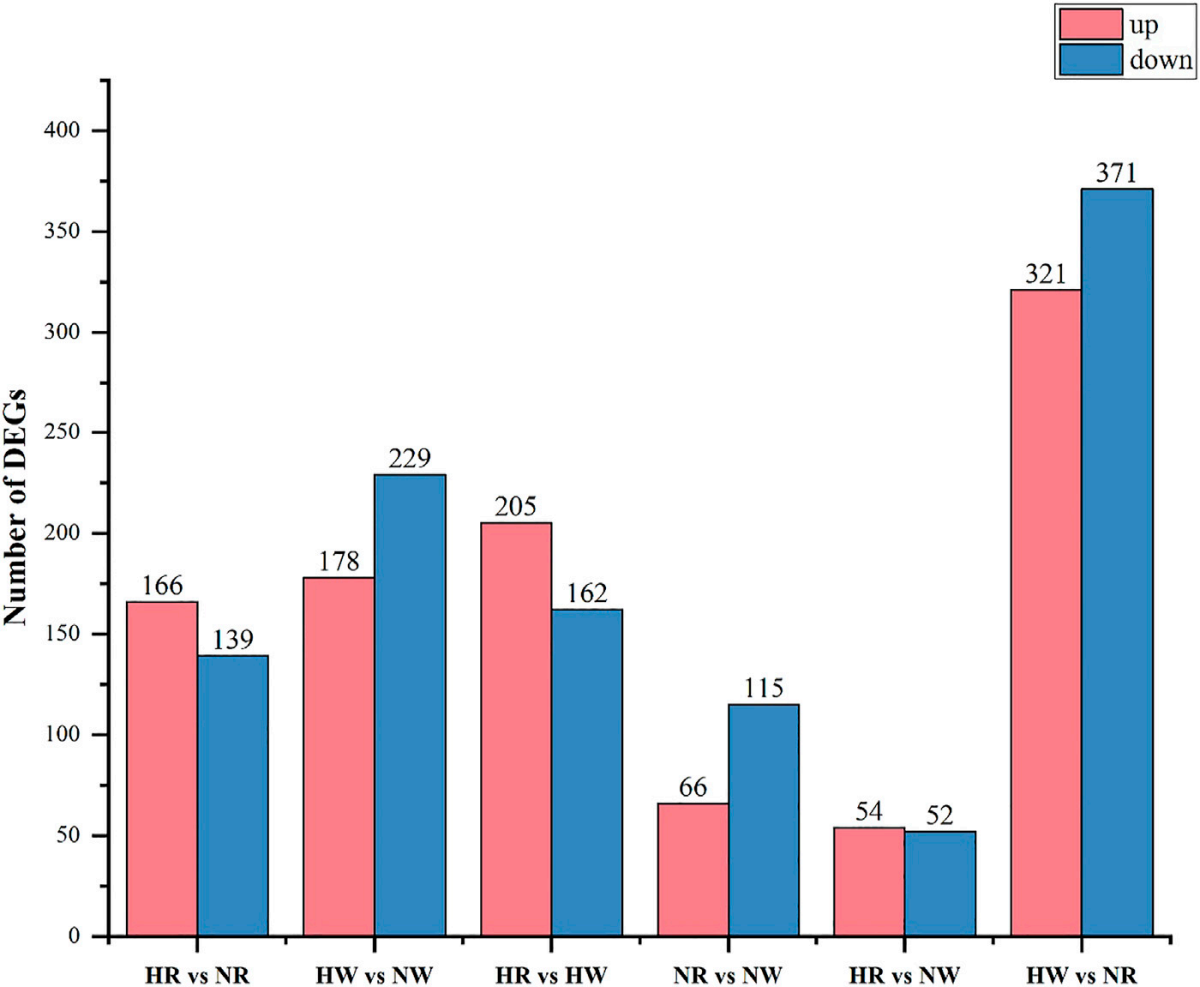
Number of DEGs in the three comparison groups.

**FIGURE 4:**
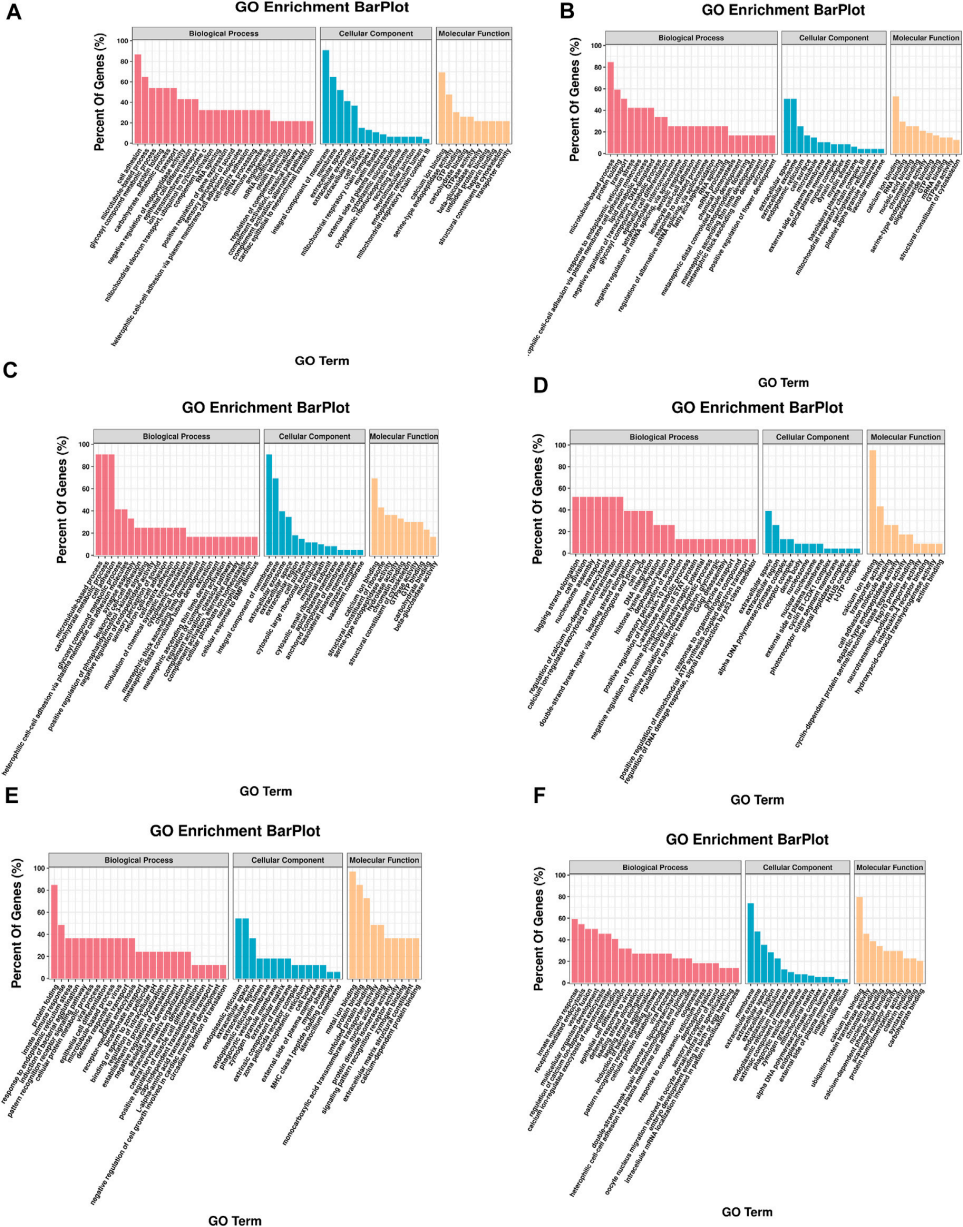
GO enrichment of DEGs. The *x*-axis is the gene functional classification of GO. The *y*-axis is the percent of DEGs in total genes in a GO term. **(A)** HR vs. NR, **(B)** HW vs. NW, **(C)** HR vs. HW, **(D)** NR vs. NW, **(E)** HR vs. NW, **(F)** HW vs. NR.

**FIGURE 5:**
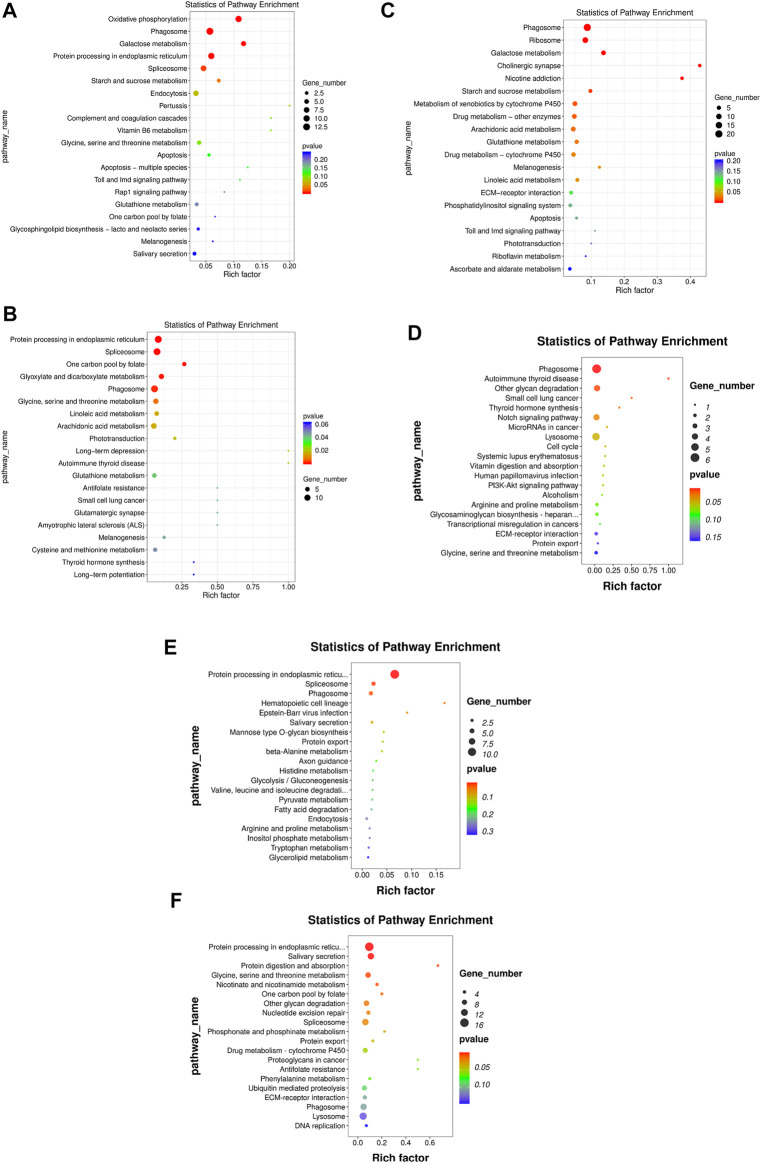
KEGG enrichment of DEGs. The *x*-axis is the rich factor, which means that the proportion of DEGs in total genes in a KEGG term. The *y*-axis is the gene functional classification of KEGG. Various colors of plots indicate different values of −log 10 (*p*-value). Plot diameter represents DEG numbers in a KEGG term (For interpretation of the references to color in this figure legend, the reader is referred to the Web version of this article.). **(A)** HR vs. NR, **(B)** HW vs. NW, **(C)** HR vs. HW, **(D)** NR vs. NW, **(E)** HR vs. NW, **(F)** HW vs. NR.

In the HR vs. NR group comparison, 305 DEGs (up-regulated: 166, down-regulated: 139) were detected (Figure 3; Supplementary Table S5), of which 227 DEGs were annotated to 1,059 gene ontology (GO) terms. The GO database was allocated among the biological processes (BP), cellular components (CC) and molecular functions (MF). “cell adhesion” “microtubule-based process” “glycosyl compound metabolic process” and “protein folding” were the most enriched subcategories under the BP (*p* < 0.05). The most enriched subcategories in CC were “integral component of membrane”, “membrane” and “extracellular space” (*p* < 0.05). The most common subcategories in MF were “calcium ion binding”, “serine-type endopeptidase activity” and “GTP binding”. The findings of the GO enrichment analyses are displayed in Figure 4A. Kyoto Encyclopedia of Genes and Genomes (KEGG) analysis revealed a total of 109 DEGs were annotated in 73 pathways. Among them, six pathways were significantly enriched, namely “phagosome (ko04145)”, “protein processing in endoplasmic reticulum (ko04141)”, “oxidative phosphorylation (ko00190)”, “galactose metabolism (ko00052)”, “spliceosome (ko03040)” and “starch and sucrose metabolism (ko00500)” (Figure 5A).

In the HW vs. NW group comparison, a total of 407 DEGs (up-regulated: 178, down-regulated: 229) were detected, of which 296 DEGs were annotated in 1059 GO terms. The GO entries that are consistent with the HR vs. NR group were “microtubule-based process”, “protein folding” and “transport”; “extracellular space”, “extracellular exosome”, “endoplasmic reticulum”; “calcium ion binding”, “structural constituent of cytoskeleton” and so on. However, the difference was that “endoplasmic reticulum stress”, “negative regulation of transcription” and “DNA-templated” were found among BP and “RNA binding”, “nuclide acid binding” and other structure-related terms were found in MF, while the DEGs were not annotated to the cell membrane entries in CC (Figure 4B). KEGG enrichment analysis showed 137 DEGs were annotation to 108 pathways, and 17 were significantly enriched (*p* < 0.05). Different from the HR vs. NR group, more basal metabolism-related pathways were annotated, such as “one carbon pool by folate (ko00670)”, “glyoxylate and dicarboxylate metabolism (ko00630)”, and “linoleic acid metabolism (ko00591)” (Figure 5B).

In the HR vs. HW group comparison, we detected 367 DEGs (up-regulated: 205, down-regulated: 162) of which there were 271 DEGs annotated to 851 GO terms. The most significantly enriched subcategories in BP, CC and MF were “microtubule-based process”, “integral component of membrane” and “calcium ion binding”, respectively (Figure 4C). KEGG analysis showed a total of 118 DEGs were annotated in 83 pathways. The significant pathways containing the most DE genes were “phagosome (ko04145)”, “ribosome (ko03010)”, “galactose metabolism (ko00052)”, “metabolism of xenobiotics (ko00980)”, “cholinergic synapse (ko04725)” and so on. The top 20 enriched pathways for each comparison are shown in Figure 5C.

In the NR vs. NW group comparison, 181 DEGs (up-regulated: 66, down-regulated: 115) were detected, of which 124 DEGs were annotated in 460 GO terms. “cell division”, “nucleosome assembly”, and “regulation of calcium ion-dependent exocytosis” were the most enriched subcategories under the BP (*p* < 0.05). The most enriched subcategories in CC were “alpha DNA polymerase: primase complex”, “dense body” and “receptor complex” (*p* < 0.05). The most common subcategories in MF were “calcium ion binding”, “Hsp70 protein binding”, “transporter activity”, “aspartic-type endopeptidase activity” and “cell adhesion molecule binding” (*p* < 0.05) (Figure 4D). KEGG enrichment analysis showed 43 DEGs were annotation to 50 pathways, and 8 were significantly enriched (*p* < 0.05), namely “phagosome”, “lysosome”, “other glycan degradation”, “notch signaling pathway”, “autoimmune thyroid disease”, “small cell lung cancer”, “thyroid hormone synthesis”, and “MicroRNAs in cancer” (Figure 5D).

In addition, we also compared HR with NW and HW with NR. In the HR vs. NW group comparison, a total of 106 DGEs were detected (up-regulated: 54, down-regulated: 52). GO enrichment analysis revealed 76 genes annotated to 337 GO terms, among which “protein folding”, “innate immune response”, “response to endoplasmic reticulum stress” were significantly enriched in BP; “endoplasmic reticulum”, “extracellular space”, “extracellular region” were significantly enriched in CC; “metal ion binding”, “protein binding”, “unfolded protein binding” were significantly enriched in MF (*p* < 0.05) (Figure 4E). KEGG analysis showed 28 DEGs were annotated in 29 pathways (Supplementary Table S6). The significant pathways containing the most DE genes were “protein processing in endoplasmic reticulum (ko04141)”, “phagosome (ko04145)”, “spliceosome (ko03040)”, and “hematopoietic cell lineage (ko04640)” (*p* < 0.05) (Figure 5E).

In the HW vs. NR group comparison, a total of 692 DGEs were detected (up-regulated: 321, down-regulated: 371). GO enrichment analysis revealed 506 genes annotated to 1401 GO terms, among which “innate immune response”, “receptor-mediated endocytosis”, “vesicle fusion” and “multicellular organism development” were significantly enriched in BP; “membrane”, “extracellular exosome”, and “extracellular space” were significantly enriched in CC; “calcium ion binding”, “ubiquitin-protein transferase activity”, and “chromatin binding” were significantly enriched in MF (*p* < 0.05) (Figure 4F). KEGG analysis showed 206 DEGs were annotated in 123 pathways (Supplementary Table S5). The significant pathways containing the most DE genes were “protein processing in endoplasmic reticulum (ko04141)”, “salivary secretion (ko04970)”, “spliceosome (ko03040)”, and “glycine, serine and threonine metabolism (ko00260)” (*p* < 0.05) (Figure 5F).

### MiRNA Profiling of *S. intermedius* in Response to High Temperature Stress

We constructed 12 miRNA libraries (NR, HR, NW, HW, n = 3) from *S. intermedius* to identify the miRNAs involved in heat stress. The numbers of raw reads of these 12 transcriptomes ranged from 10 to 30 million (Bioproject ID: PRJNA783268). After filtering for reads with 3′ adapters and length filters, junk reads, Rfam, mRNA, repeats, rRNA, tRNA, snRNA and snoRNA sequences, as well as other Rfam RNA sequences (see Methods), we obtained 124,484 to 704,950 unique sequences from 12 miRNA libraries (Supplementary Table S7). Most of the clean reads were 21–23 nt in length, with 22 nt being the most abundant length in all libraries (Supplementary Figure S1).

**FIGURE 6:**
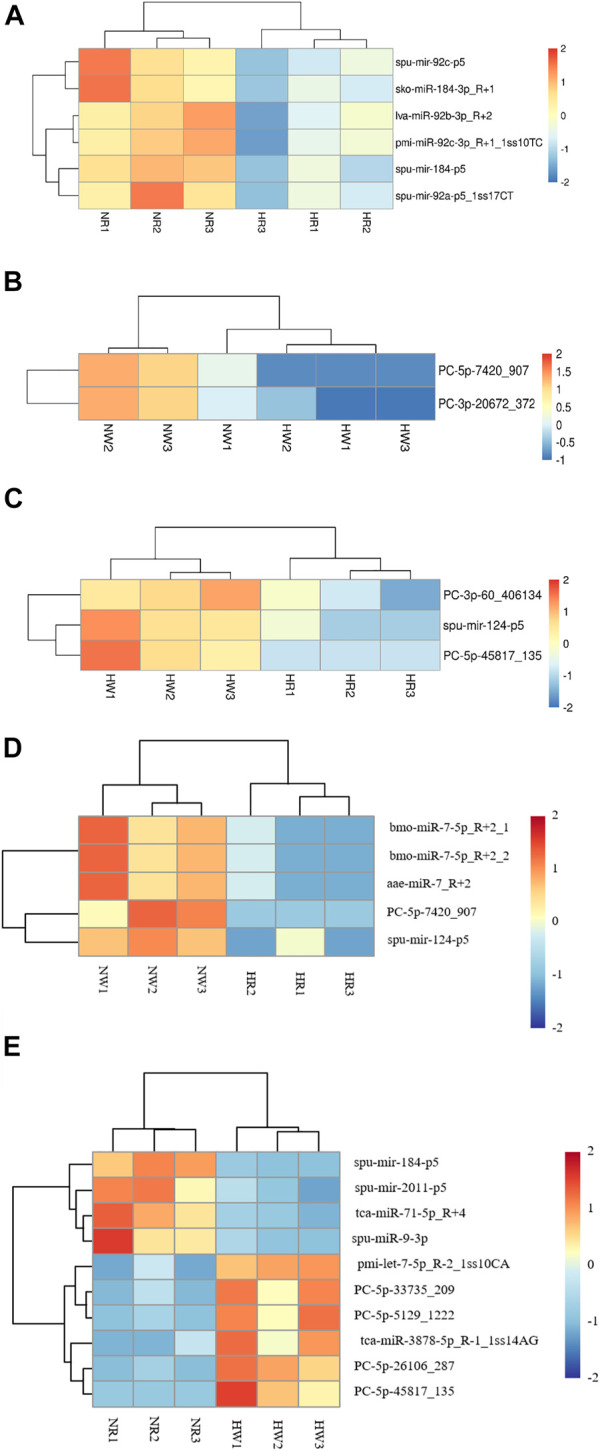
Heat map of three groups of DE miRNA clustering analysis. The *x*-axis are samples and the *y*-axis are miRNAs. Different colors indicate different miRNA expression levels, and colors from blue via white to red indicate expression (log10 (norm value)) from low to high. Red color indicates high expression miRNA and dark blue color indicates low expression miRNA. **(A)** HR vs. NR, **(B)** HW vs. NW, **(C)** HR vs. HW, **(D)** HR vs. NW, **(E)** HW vs. NR.

Based on bioinformatic analysis, 671 miRNAs were identified; 195 known miRNAs and 476 novel miRNAs were expressed in all samples (Table 1). By co-expression analysis of four library groups (six comparison groups), a total of 90 (55 co-expressed, HR: 1, NR: 34), 156 (120 co-expressed, HW: 16, NW: 20), 137 (58 co-expressed, HR: 1, HW: 78), 143 (86 co-expressed, NR: 3, NW: 54), 143 (86 co-expressed, NW: 54, NR: 3), 141 (58 co-expressed, HR: 1, NW: 82), 139 (86 co-expressed, HW: 50, NR: 3) miRNAs were identified in the HR vs. NR, HW vs. NW, HR vs. HW, NR vs. NW, HR vs. NW, and HW vs. NR groups, respectively (Supplementary Figure S2). We then explored differentially expressed miRNAs (DE miRNAs) in the different comparison groups. Six (miR-184-p5, miR-184-3p_R+1, miR-92a-p5_lss17CT, miR-92b-3p_R+2, miR-92c-p5, and miR-92c-3p_R+1_lss10TC), two (PC-5p-7420_907, PC-3p-20672_372), three (miR-124-p5, PC-3p-60_406134, PC-5p-45817_135) DE miRNAs, one (PC-5p-7420_907), five (miR-7-5p_R+2_2, miR-7_R+2, miR-7-5p_R+2_1, PC-5p-7420_907, and miR-124-p5), and ten (miR-184-p5, PC-5p-26106_287, miR-71-5p_R+4, miR-2011-p5, PC-5p-33735_209, miR-3878-5p_R-1_1ss14AG, PC-5p-5129_1,222, pmi-let-7-5p_R-2_1ss10CA, miR-9-3p, and PC-5p-45817_135) DE miRNAs were identified in pairwise comparisons of HR vs. NR, HW vs. NW, HR vs. HW, NR vs. NW, HR vs. NW, and HW vs. NR respectively. The heatmaps of DE miRNAs for the remaining comparison groups were shown in Figure 6, except for the NR vs. NW group.

**TABLE 1:** Number of known and novel miRNA identified in each sample.

	NR Library					
	NR1		NR2		NR3	
Groups	Pre-miRNA	Unique miRNA	Pre-miRNA	Unique miRNA	Pre-miRNA	Unique miRNA
gp1	33	46	39	68	38	63
gp2a	14	13	20	19	19	18
gp2b	6	6	17	16	17	17
gp3	15	17	35	38	31	34
gp4	96	90	64	56	76	66
	NW library					
	NW1		NW2		NW3	
	Pre-miRNA	Unique miRNA	Pre-miRNA	Unique miRNA	Pre-miRNA	Unique miRNA
gp1	36	59	40	65	38	62
gp2a	19	18	19	17	20	19
gp2b	16	15	19	19	16	16
gp3	32	35	39	41	36	39
gp4	68	58	78	73	81	69
	HR library					
	HR1		HR2		HR3	
Groups	Pre-miRNA	Unique miRNA	Pre-miRNA	Unique miRNA	Pre-miRNA	Unique miRNA
gp1	38	62	40	67	39	64
gp2a	20	19	19	18	20	18
gp2b	16	16	18	17	12	12
gp3	36	39	43	45	35	38
gp4	81	69	63	71	60	34
	HW library					
	HW1		HW2		HW3	
Groups	Pre-miRNA	Unique miRNA	Pre-miRNA	Unique miRNA	Pre-miRNA	Unique miRNA
gp1	30	31	39	62	39	63
gp2a	7	6	19	18	21	18
gp2b	6	6	12	12	11	16
gp3	10	11	29	32	36	40
gp4	31	76	65	58	53	66

### Identification and Functional Annotation of the Target Genes of the DE miRNAs

**FIGURE 7:**
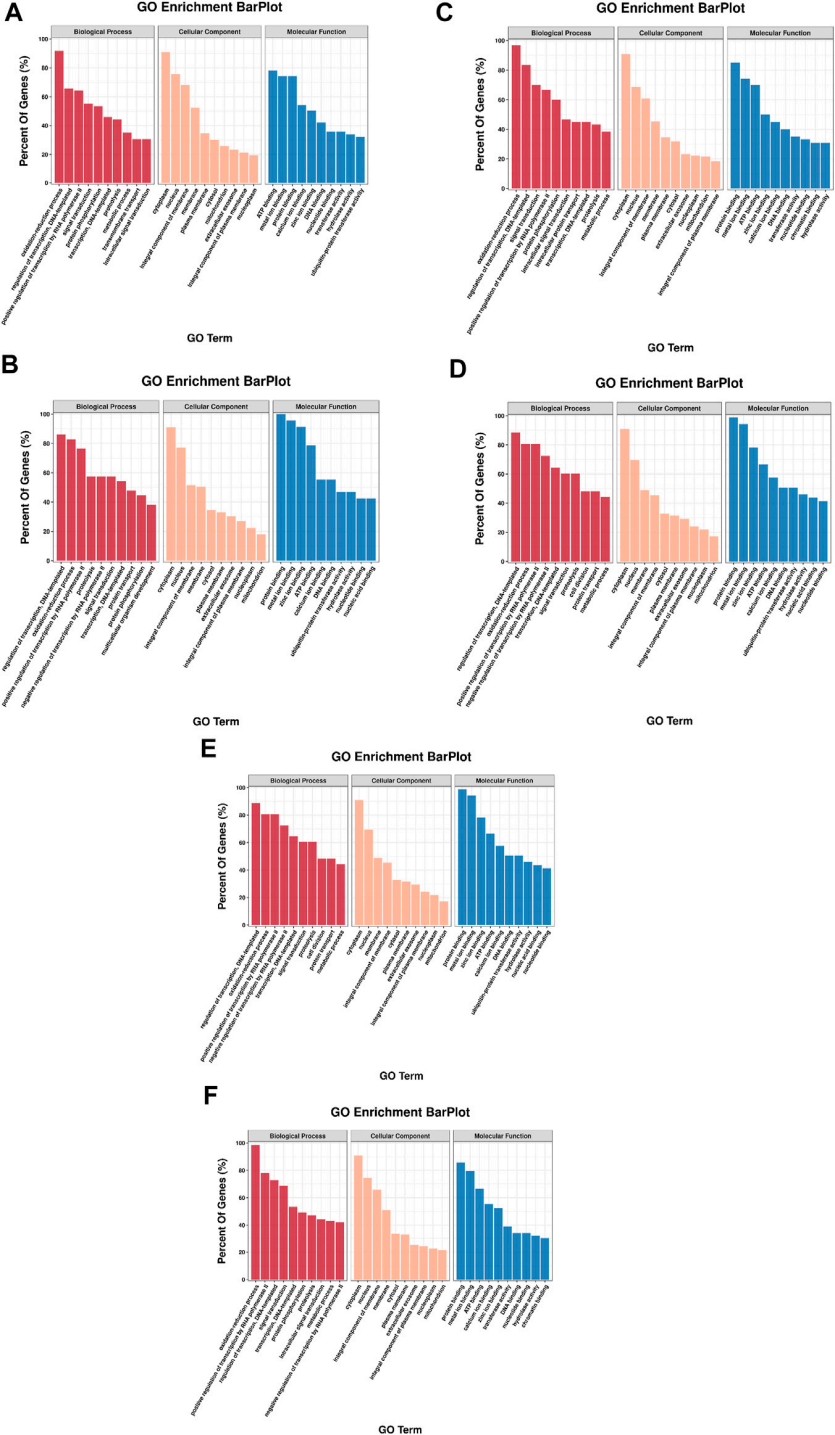
GO annotation of DE miRNA target genes in each comparison group. The top 10 GO terms were shown in each class. **(A)** HR vs. NR, **(B)** HW vs. NW, **(C)** HR vs. HW, **(D)** NR vs. NW, **(E)** HR vs. NW, **(F)** HW vs. NR.

To explore DE miRNAs in the different comparison groups and further understand the regulatory functions of the miRNAs, we screened DE miRNAs and performed GO and KEGG enrichment analysis on the predicted target genes. A total of 2,543, 1,190, 1744, 984, 3,614, and 5,345 target genes were predicted for the six, two, three, one, five, and ten DE miRNAs identified from the six comparison groups, respectively (Supplementary Table S8), and GO analysis was used to identify enriched functional groups (*p* < 0.05). The top 10 significantly enriched GO terms (with lowest *p*-values) of the three categories are shown in Figure 7. Among the three groups, “regulation of transcription, DNA-templated”, “oxidation-reduction process”, “cytoplasm”, “nucleus”, “ATP binding” and “protein binding” were the entries with the highest significant enrichment of target genes in BP, CC and MF.

**FIGURE 8:**
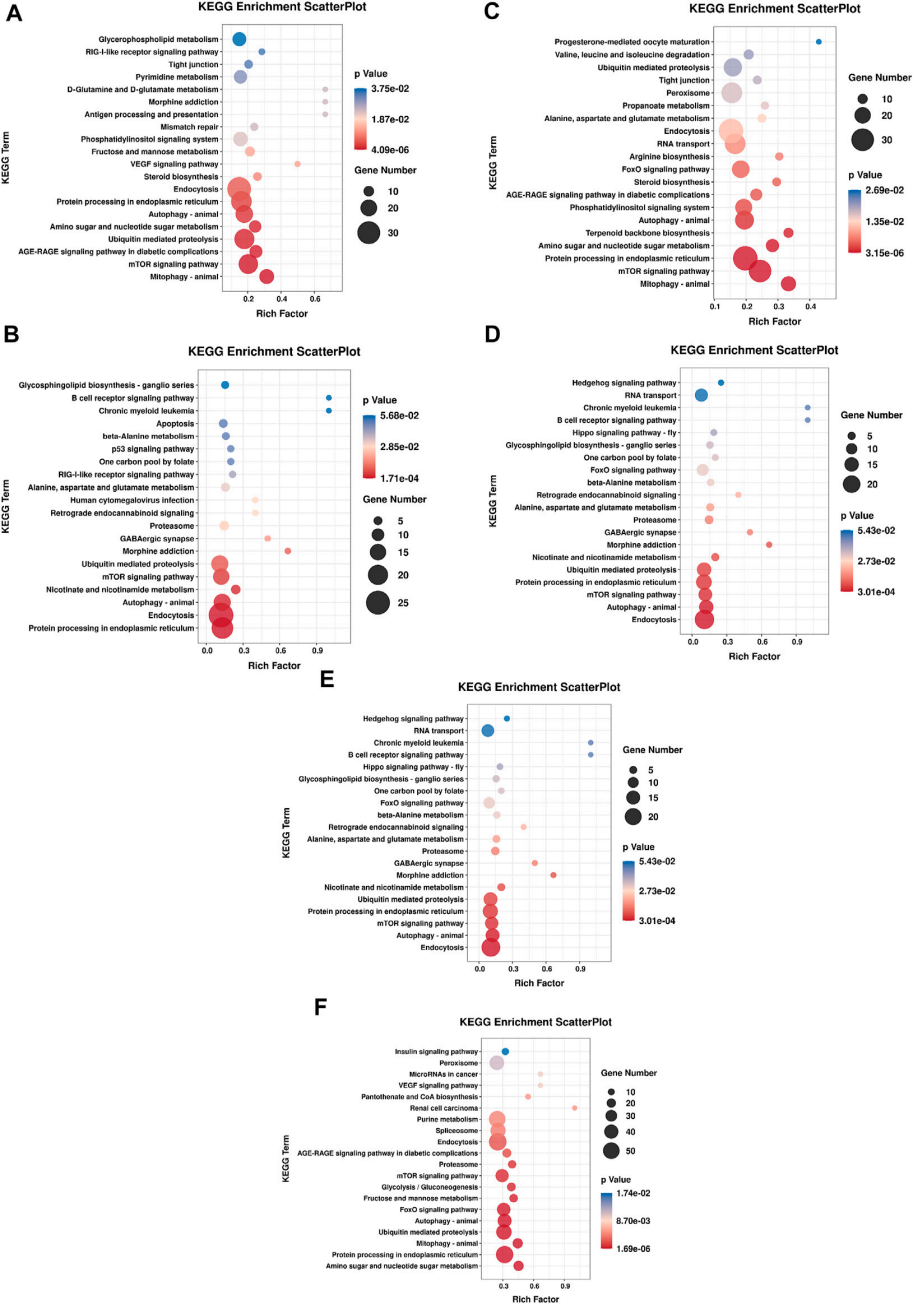
Enrichment analysis of KEGG pathway of DE miRNA targets in each comparison group. The top 20 enriched pathways were shown in each graph. **(A)** HR vs. NR, **(B)** HW vs. NW, **(C)** HR vs. HW, **(D)** NR vs. NW, **(E)** HR vs. NW, **(F)** HW vs. NR.

KEGG pathway annotation showed 604, 316, 196, 316, 629 and 1,117 predicted target genes were annotated into 193, 161, 128, 161, 200, and 233 pathways, respectively (*p* < 0.05). Among them, more target genes were enriched in the classes of cellular processes, environmental information processing and metabolism. KEGG pathway analysis based on all of the predicted target genes revealed that DE miRNAs were involved in several pathways relevant to sea urchins exposed to high temperature, including protein processing in the endoplasmic reticulum (ko04144) and ubiquitin mediated proteolysis (ko04120), which are related to protein synthesis, and amino sugar and nucleotide sugar metabolism (ko00520) and alanine, aspartate and glutamate metabolism (ko00250), which are involved in basic metabolism. Furthermore, several immune-related pathways have also been discovered, including phagosome (ko04145), endocytosis (ko04144), autophagy (ko04140), mTOR signaling (ko04150) and FoxO signaling (ko04086) (Figure 8).

### Prediction of Interactions Between DE miRNAs and DEGs

**FIGURE 9:**
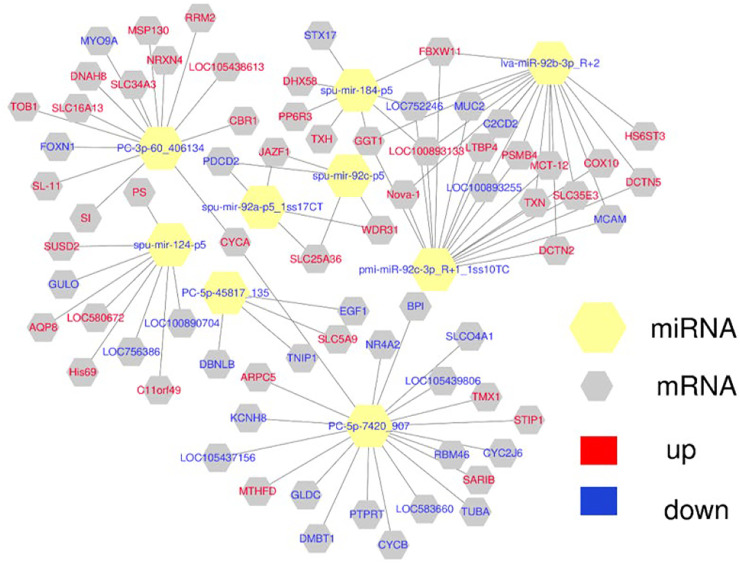
The network of DE miRNA and their target DEGs was constructed using Cytoscape 3.3.0. Yellow hexagon is miRNA, gray hexagon is target DEGs, red represents up-regulation, blue represents down-regulation.

Combining the results for the DE miRNAs target genes and DEGs, we found that 5 DE miRNAs may have targeted regulatory relationships with 28 DEGs in the HR vs. NR group. Among these, 35 “DE miRNA-DEG” pairs exhibited a negative expression correlation, whereas 17 “DE miRNA-DEG” pairs showed a positive expression correlation. In the HW vs. NW group, we found that 1 DE miRNA may have targeted regulatory relationships with 20 DEGs. Among these, a negative expression correlation was found for 5 “DE miRNA-DEG” combinations, while a positive expression correlation was found for 15 “DE miRNA-DEG” pairs. In the HR vs. HW group, 3 DE miRNAs may have targeted regulatory connections with 27 DEGs. There was a negative expression correlation for 19 “DE miRNA-DEG” pairs, whereas there was a positive expression correlation for 8 “DE miRNA-DEG” pairs. In the NR vs. NW group, 1 DE miRNAs may have targeted regulatory connections with 10 DEGs. Among these, a negative expression correlation was found for 2 “DE miRNA-DEG” combinations, while a positive expression correlation was found for 8 “DE miRNA-DEG” pairs. In the HR vs. NW group, 5 DE miRNAs may have targeted regulatory connections with 16 DEGs. There was a negative expression correlation for 17 “DE miRNA-DEG” pairs, whereas there was a positive expression correlation for 18 “DE miRNA-DEG” pairs. In the HW vs. NR group, 10 DE miRNAs may have targeted regulatory connections with 107 DEGs. There was a negative expression correlation for 94 “DE miRNA-DEG” pairs, whereas there was a positive expression correlation for 70 “DE miRNA-DEG” pairs. This study focused on the response of sea urchins to high temperature, so we constructed mRNA-miRNA network maps for HR vs. NR, HW vs. NW, HR vs. HW groups. (Figure 9 and Supplementary Table S9).

**FIGURE 10:**
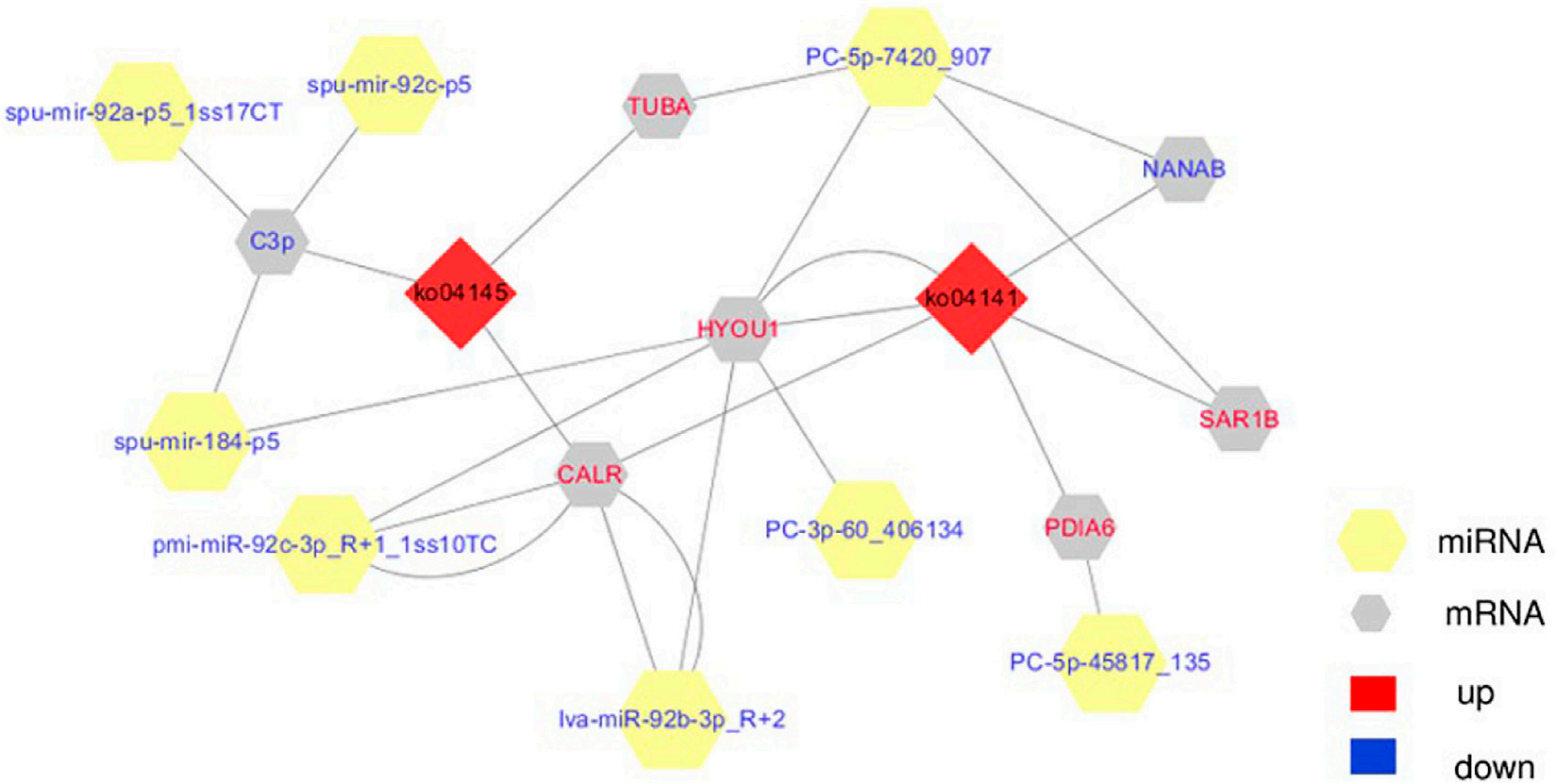
The integrating biomolecular interaction network of target DEGs predicted to be regulated by DE miRNA in the “protein processing in endoplasmic reticulum (ko04141)” and “phagosome (ko04145)” pathways. Red diamond is KEGG pathway, yellow hexagon is miRNA, gray hexagon is target DEGs, red represents up-regulation, blue represents down-regulation.

Since transcriptional attenuation of target genes is a conserved mechanism of miRNA regulation, we only focused on DEGs and DE miRNAs with negative regulatory relationships. For the “protein processing in endoplasmic reticulum (ko04141)" and “phagosome (ko04145)" pathways, we used Cytoscape software to map the network of DE miRNAs and targeted DEGs of these two pathways and found that miR-92b-3p and PC-5p-7420 were the most connected miRNAs, and lva-miR-92b-3p_R+2 and pmi-miR-92c-3p_R+1_1ss10TC co-regulate *CALR* (Table 2; Figure 10).

**TABLE 2:** “DE miRNA- DEG” pairs in the “protein processing in endoplasmic reticulum” and “phagosome” pathways.

miRNA ID	miRNA	DEGs Name	Transcript ID	DEGs Pvalue	DEGs Up/Down	Discription	KEGG Pathway	Regulation
	Up/Down							
PC-3p-60_406134	down	*HYOU1*	EVM0021577.4	0.03	up	PREDICTED: hypoxia up-regulated protein 1 [*Strongylocentrotus purpuratus*)	ko04141(Protein processing in endoplasmic reticulum)	−
lva-miR-92b-3p_R+2	down	*CALR*	EVM0001684.10	0	up	calreticulin precursor (*Strongylocentrotus purpuratus*)	ko04141(Protein processing in endoplasmic reticulum); ko04145(Phagosome)	−
pmi-miR-92c-3p_R+1_1ss10TC	down	*CALR*	EVM0001684.10	0	up	calreticulin precursor (*Strongylocentrotus purpuratus*)	ko04141(Protein processing in endoplasmic reticulum); ko04145(Phagosome)	−
PC-5p-45817_135	down	*PDIA6*	EVM0010502.1	0	up	PREDICTED: probable protein disulfide-isomerase A6 (*Strongylocentrotus purpuratus*)	ko04141(Protein processing in endoplasmic reticulum)	−
PC-5p-7420_907	down	*SAR1B*	EVM0013723.1	0.03	up	PREDICTED: GTP-binding protein SAR1b isoform X1 (*Strongylocentrotus purpuratus*)	ko04141(Protein processing in endoplasmic reticulum)	−
PC-5p-7420_907	down	*NANAB*	EVM0021503.1	0.03	down	PREDICTED: LOW QUALITY PROTEIN: neutral alpha-glucosidase AB (*Strongylocentrotus purpuratus*)	ko00510(N-Glycan biosynthesis); ko04141(Protein processing in endoplasmic reticulum)	+
PC-5p-7420_907	down	*HYOU1*	EVM0021577.4	0.03	up	PREDICTED: hypoxia up-regulated protein 1 (*Strongylocentrotus purpuratus*)	ko04141(Protein processing in endoplasmic reticulum)	−
lva-miR-92b-3p_R+2	down	*HYOU1*	EVM0021577.4	0.03	up	PREDICTED: hypoxia up-regulated protein 1 (*Strongylocentrotus purpuratus*)	ko04141(Protein processing in endoplasmic reticulum)	−
pmi-miR-92c-3p_R+1_1ss10TC	down	*HYOU1*	EVM0021577.4	0.03	up	PREDICTED: hypoxia up-regulated protein 1 (*Strongylocentrotus purpuratus*)	ko04141(Protein processing in endoplasmic reticulum)	−
spu-mir-184-p5	down	*HYOU1*	EVM0021577.4	0.03	up	PREDICTED: hypoxia up-regulated protein 1 (*Strongylocentrotus purpuratus*)	ko04141(Protein processing in endoplasmic reticulum)	−
lva-miR-92b-3p_R+2	down	*CALR*	EVM0001684.10	0	up	calreticulin precursor (*Strongylocentrotus purpuratus*)	ko04141(Protein processing in endoplasmic reticulum); ko04145(Phagosome)	−
pmi-miR-92c-3p_R+1_1ss10TC	down	*CALR*	EVM0001684.10	0	up	calreticulin precursor (*Strongylocentrotus purpuratus*)	ko04141(Protein processing in endoplasmic reticulum); ko04145(Phagosome)	−
spu-mir-184-p5	down	*C3p*	EVM0004046.1	0.01	down	complement component C3 precursor (*Strongylocentrotus purpuratus*)	ko04145(Phagosome)	+
spu-mir-92a-p5_1ss17CT	down	*C3p*	EVM0004046.1	0.01	down	complement component C3 precursor (*Strongylocentrotus purpuratus*)	ko04145(Phagosome)	+
spu-mir-92c-p5	down	*C3p*	EVM0004046.1	0.01	down	complement component C3 precursor (*Strongylocentrotus purpuratus*)	ko04145(Phagosome)	+
PC-5p-7420_907	down	*TUBA*	EVM0013887.1	0.02	up	Tubulin alpha-1B chain (*Scophthalmus maximus*)	ko04145(Phagosome); ko04210 (Apoptosis); ko04530(Tight junction); ko04540(Gap junction)	−
PC-3p-60_406134	down	*HYOU1*	EVM0021577.4	0.03	up	PREDICTED: hypoxia up-regulated protein 1 (*Strongylocentrotus purpuratus*)	ko04141(Protein processing in endoplasmic reticulum)	−

### Validation of DEGs and DE miRNAs by qRT-PCR

**FIGURE 11:**
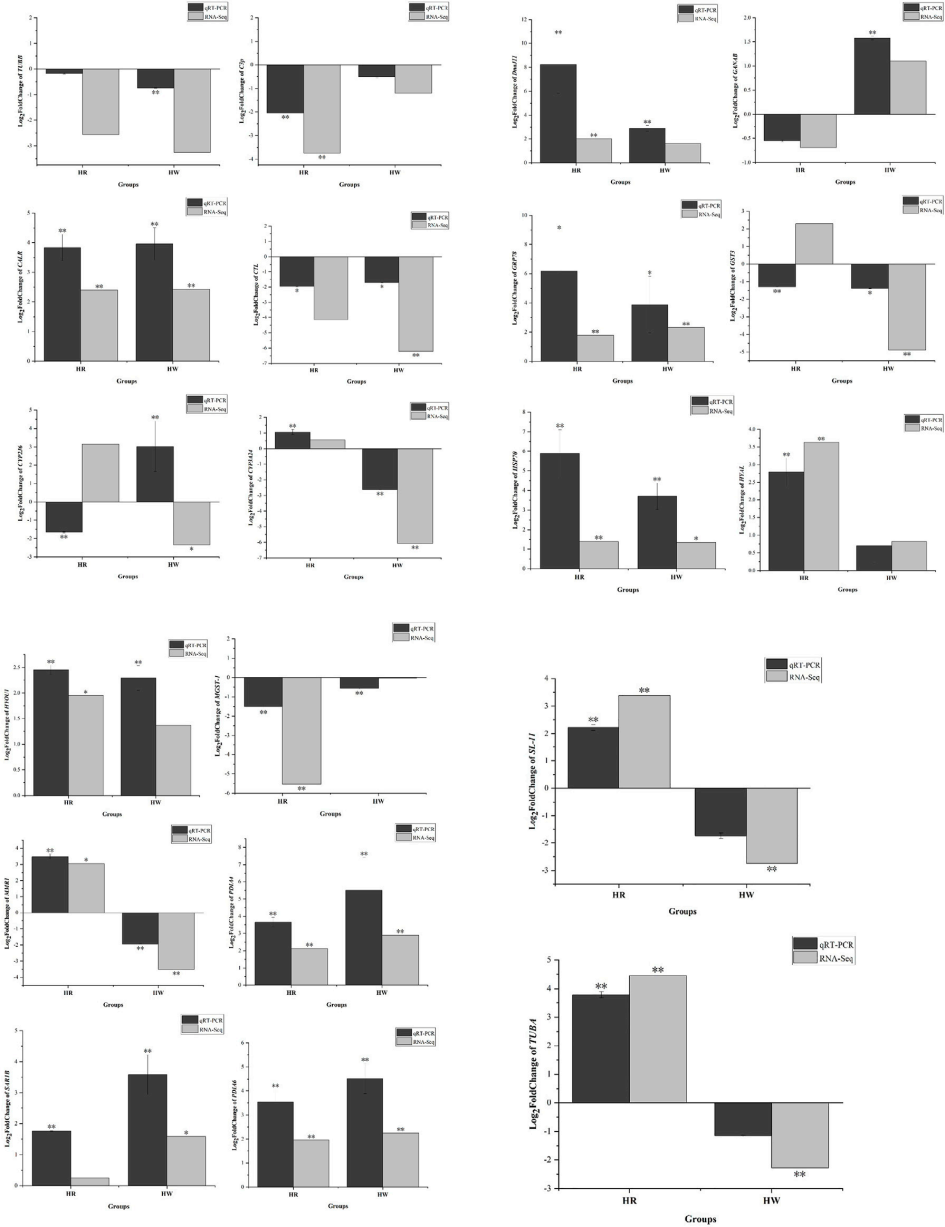
Analysis of selected 20 differentially expressed genes (DEGs) in red and white tube-footed *S. intermedius* under the high temperature stress by sequencing and qRT-PCR. Each vertical bar represents the Mean ± SD (n = 3), *18s rRNA* were used as a reference gene. *Significant differences at *p* < 0.05 vs. control (NR and NW). **Highly significant differences at *p* < 0.01 vs. control (NR and NW).

**FIGURE 12:**
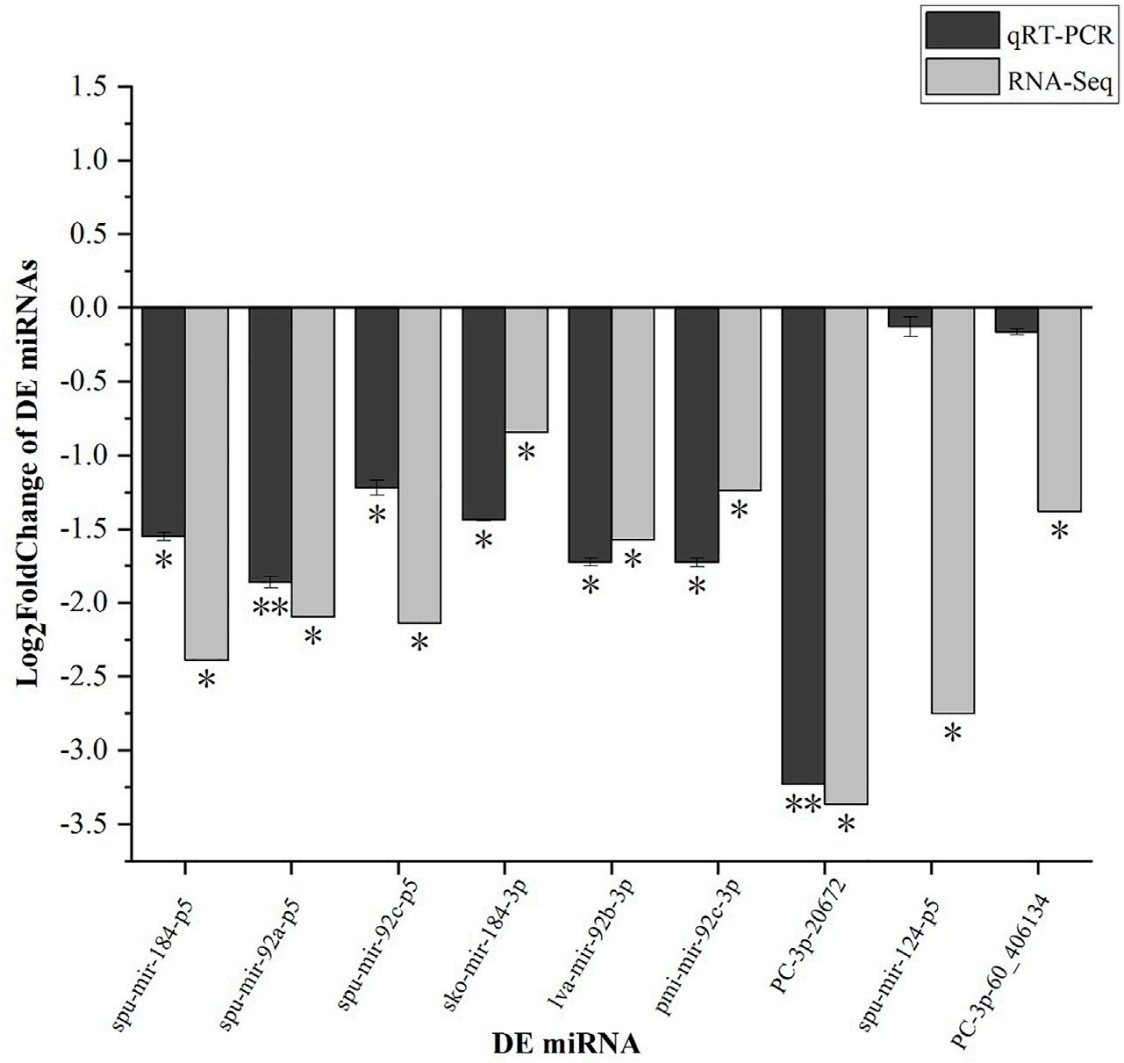
qRT-PCR verification of select 9 DEMs. Each vertical bar represents the Mean ± SD (n = 3), *18s rRNA* were used as a reference gene. *Significant differences at *p* < 0.05 vs. control (NR or NW). **Highly significant differences at *p* < 0.01 vs. control (NR or NW).

qRT-PCR analysis was performed to validate the results of the differential gene expressions obtained from the RNA-Seq data. A total of 20 DEGs (such as *HSP70*, *DnaJ11*, and *CALR*) and 9 DE miRNAs were selected for the qRT-PCR measurements. The results of qRT-PCR analysis coincided with the results generated from RNA-Seq and showed similar expression trends to those of the RNA-Seq data (Figures 11, 12). In addition, qRT-PCR results showed that the expression of *HSP70*, *DnaJ11*, *GRP78*, *HYAL*, and *HYOU1* was significantly enhanced under high temperature stress, and the expression was higher in the red tube-footed sea urchin than in the white tube-footed sea urchin.

## Discussion

Temperature is the most important environmental element impacting organisms, and it can alter physiological and metabolic processes directly or indirectly. Mariculture organisms are primarily found in environmentally fragile coastal zones or estuaries, which are particularly vulnerable to environmental change (Dong et al., 2015; Ma et al., 2021). The *S. intermedius* is highly sensitive to environmental changes as a cold-water species, and significant temperature changes are one of the leading causes of mass mortality of sea urchins in China during the summer. Published biological and ecological data on the morphological forms of *S. intermedius* indicated that milk-white color of spines (G) prevails in deep-water settlements (15–25 m) and the “usual” (U) form that mostly occurs in shallow-water settlements (5–10 m) (Balakirev et al., 2008; Balakirev et al., 2016). The milk-white color form preferring deep water conditions indeed could be less tolerant to high-temperature stress because the temperature fluctuations are much more significant in shallow-water than in deep-water environments (especially in the summer time). This study was conducted to investigate the changes in antioxidant enzyme activity under high temperature stress and to explore the candidate genes and regulatory pathways of *S. intermedius* in response to high temperature stress using RNA-Seq technology in order to better understand the molecular mechanisms invoked by sea urchins in response to high temperature stress.

In response to environmental stress, organisms usually activate humoral immunity and alter their antioxidant enzyme activity to buffer the effects of temperature changes on the organism (Lu et al., 2016; Forgati et al., 2017). The antioxidant system, which primarily acts through antioxidant enzymes (such as SOD and CAT) and non-enzymatic antioxidants, protects organisms from oxidative damage (such as GSH). SOD is the first stage in the antioxidant process (Winston, 1991; Grundy and Storey, 1998), and its activity refers to the ability of an organism to scavenge free radicals, which is a key sign of its health (Luo et al., 2017). In this study, we evaluated the enzyme activities after 7 days of stress at 25°C. Compared with the control group at 15°C, the SOD, POD and GSH-Px activities of red and white tube-footed sea urchins were elevated, and the SOD activity of red tube-footed sea urchins was higher than that of white tube-footed sea urchins and the changes in their POD activity were significantly different (*p* < 0.05). This finding is consistent with the study of Ding et al. (2015), who investigated the effect of temperature on the activity of immune-related enzymes in *S. intermedius* with different colored tube feet, and our results confirmed the argument that the SOD activity of red tube-footed sea urchins responded to high temperature earlier than that of white tube-footed sea urchins. Meanwhile, we found no significant difference in CAT activity of the sea urchins under high temperature stress, suggesting that sea urchins use SOD more to regulate their ability to scavenge free radicals, or perhaps that enzyme activity is chronological and the magnitude of enzyme activity is related to sampling time. Therefore, we suggested that at the physiological-ecological level, red tube-footed sea urchins are more sensitive to high temperatures and have the potential to tolerate high temperatures.

In order to investigate the molecular mechanisms of sea urchins with varied tube foot colors in response to high temperature stress, gene expression profiles and miRNA profiles of *S. intermedius* with different tube foot colors were constructed in this study. Compared to previous studies (Ding et al., 2017; Zhao C et al., 2018), this study used our own sequenced genome of *S. intermedius* to construct a higher quality of data with the reference transcriptome and screened for more DEGs. We found that under normal rearing, the immune-related pathways such as “phagosome” and “lysosome” were significantly enriched in the red tube-footed *S. intermedius*. We found the number of DEGs in red tube-footed sea urchins was less than that in white tube-footed sea urchins under high temperature stress compared with the control groups. This finding is in accordance with earlier research (Shi et al., 2020). 20 DEGs and 9 DE miRNAs were screened for qRT-PCR analysis to validate the RNA-Seq results, and the results showed that the qRT-PCR expression trends were consistent with the RNA-Seq data, validating the reproducibility of our RNA-Seq research.

The “protein processing in endoplasmic reticulum” pathway, which alters protein folding in the endoplasmic reticulum, was the pathway most affected by high temperature stress (Li et al., 2017), according to the KEGG enrichment analysis of DEGs in the HR vs. NR and HW vs. NW groups. The expressions of *HSP70*, *GRP78* and *PDIA6* were considerably elevated under high temperature stress, showing that heat stress causes protein misfolding and triggers the stress response. This result is consistent with the results of Yang et al. (2017), who studied the heat stress response mechanism in oysters (*Crassostrea gigas*). Heat shock proteins are chaperone proteins that promote the degradation of abnormal proteins or reactivate stress-damaged proteins by promoting the proper refolding of denatured proteins (Morimoto et al., 1992; Zhao Z. W et al., 2018). The increased expression of *HSP70* would improve the thermal tolerance of cells under high temperature induction, limiting the production of oxygen radicals caused by heat stress and thereby reducing the rate of heat stress-related cell death (Zhou et al., 2004). Previous research has found that *S. intermedius HSP70* responds to rapid temperature changes in a variety of tissues (coelomocyte, tube feet, intestine, gonads) with differential expression and that *HSP70* expression increases and then decreases with stress time (Bai et al., 2015). *GRP78* (glucose-regulated protein 78 kDa) is a member of the *HSP70* family, and it is a key indicator of endoplasmic reticulum stress (Lee, 2005). This reveals that these DEGs may help misfolded proteins refold into the right molecular shape. The expressions of essential unfolded protein response (UPR) components including calreticulin (*CALR*) and protein disulfide isomerase (*PDI*) were also shown to be significantly up-regulated. *CALR* is a Ca^2+^-binding protein that regulates intracellular Ca^2+^ dynamic homeostasis and signaling activities such as the UPR and related protein degradation pathways, as well as regulating the immunological response, shielding cells from stress and regulating the cell cycle (Eggleton et al., 2016). *PDI* promotes protein folding by acting as a chaperone molecule (Schröder and Kaufman, 2005). Ding et al. discovered 18 distinct immune-related pathways in the transcriptome of red tube-footed sea urchins when compared to white tube-footed sea urchins, implying that red tube-footed sea urchins have a stronger immune response than white tube-footed sea urchins (Ding et al., 2017). We also discovered 13 potentially specific KEGG pathways of red tube-footed sea urchins under high temperature stress, including “phagosome”, “ribosome” and “cholinergic synapse”, as well as other immune-related and basal metabolic pathways such as “gluconeogenesis”. The immunological response and basal metabolism of red tube-footed sea urchins are thought to be boosted in high-temperature environments to protect the organism from stress damage. It can thus be suggested that red tube-footed sea urchins are more sensitive than white tube-footed sea urchins to respond to heat stress.

Gene expression is thought to be influenced by a variety of factors. Recently, researchers have investigated changed gene expression at the post-transcriptional stage in a variety of biological systems using miRNA-mRNA regulatory networks (Zhou et al., 2019; Wang et al., 2020). The miRNA profiles of the sea urchin *S. intermedius* under temperature stress are presented in this study. The experimental results demonstrated that miRNAs are mostly 22 nt in length, which is consistent with earlier research (Zhan et al., 2018). The 11 identified DE-miRNAs were down-regulated and involved in multiple key biological processes related to biosynthesis, metabolism, immunity and signaling transduction.

Since transcriptional attenuation of target genes is a conserved mechanism of miRNA regulation, we only focused on “DEG-DE miRNA” pairs with opposite relative expression trends in this study. After screening, we found that these “DEG-DE miRNA” pairs may explain the superiority of energy metabolism and immunity of red tube-footed sea urchins in response to high temperature stress. It has been shown that miR-184 is an important regulator of stem cell proliferation and growth (Liu et al., 2010), and that its overexpression can lead to apoptosis and its suppression can increase cell numbers (Foley et al., 2010). Previous studies have shown that miR-184 overexpression inhibits autophagy and exacerbates oxidative damage, and it can also negatively regulate Wnt signaling *in vivo* and *in vitro* (Liu et al., 2010). MiR-184 could inhibit protein expression in human trabecular meshwork cell cytotoxicity, apoptosis and the extracellular matrix via targeting *HIF-1α in vivo*, and it can also exhibit angiostatic properties through regulating signaling pathways including *Akt*, *TNF-α* and *VEGF* (Takahashi et al., 2015). The target gene of miR-184 was discovered to be hypoxia up-regulated protein 1 (*HYOU1*) in this study, and the two had a negative regulatory connection. When cells are stressed by the external environment (hypoxia, ischemia and high temperature etc.), *HYOU1*, as an endoplasmic reticulum molecular chaperone, protects and maintains cell viability (Park et al., 2017). Therefore, we hypothesized that miR-184 regulates the expression of genes involved in cell proliferation and autophagy in red tube-footed sea urchins in response to heat stress. We also found that red tube-footed sea urchins displayed down-regulation of miR-92a, miR-92c and miR-92b under high temperature stress. MiR-92a belongs to the miR-17–92 cluster, which has six mature miRNAs (Tsuchida et al., 2011). Members of this cluster are involved in the development of vascular endothelial cells and regulate numerous essential biological processes such as cell proliferation and death (Tamatani et al., 2001; Olive et al., 2009; Olive et al., 2010; Kaluza et al., 2013; Tan et al., 2014). Additionally, miR-92a promotes cell proliferation by inhibiting an isoform of the cell cycle regulator p63 (Manni et al., 2009). Yang et al. also showed that miR-92d can regulate the complement system in the amphioxus *Branchiostoma belcheri* to modulate the acute immunological response to bacterial infections (Yang et al., 2013). Lv et al. reported miR-92a is involved in apoptotic signaling pathway regulation perhaps via targeting *Aj14-3-3ζ* in sea cucumbers (Lv et al., 2017). MiR-92c and miR-92b were discovered to influence *CRT* expression in our study, and more research will be done to confirm this. We also discovered miR-124 by comparing the miRNA expression profiles of red and white tube-footed sea urchins at high temperatures. In the mouse brain, miR-124 promotes neuronal development by triggering particular alternative pre-mRNA splicing (Makeyev et al., 2007). Non-neuronal transcripts are increased in primary cortical neurons when miR-124 is inhibited (Conaco et al., 2006). We hypothesized that decreased miR-124 expression led to a stimulation of neural differentiation in red tube-footed sea urchins under high temperature stress, making them more temperature sensitive and thus faster to respond to high temperature stress. Two new miRNAs, PC-3p-45817 and PC-5p-7420, were identified by association analysis, in which the target genes of PC-2p-4581 were *DNAJ11* and *PDIA6*, and the target genes of PC-5p-7420 were *GANAB*, *SAR1B* and *TUBA*. These genes play an important role in the UPR response to endoplasmic reticulum stress (Gotoh et al., 2004; Sha et al., 2012). Our findings support the idea that miRNA-target gene interactions are complicated, and that a single miRNA can regulate multiple target genes simultaneously, whereas a single gene can be regulated by multiple miRNAs at the same time (Lan et al., 2016). The results of the qRT-PCR validation further imply that there may be meaningful targeted regulatory interactions between th candidate DE miRNAs and DEGs. As a consequence, in future research, we will need to confirm the targeted regulatory interactions of these putative “DEG-DE miRNA” pairs utilizing additional *in vivo* and *in vitro* experiments (e.g., dual luciferase reporter analysis, gain or loss of function analysis and Western blotting).

## Conclusion

To research the molecular processes involved in the sea urchin response to high thermal stress, we investigated gene expression differences and miRNA profiles of red and white tube-footed sea urchins under high temperature stress conditions. According to previous studies and miRNA-mRNA integration analysis, protein folding of the endoplasmic reticulum and phagosomes are the main pathways for sea urchins to respond to high temperature stress, and red tube-footed sea urchins may be more sensitive to high temperature and respond more quickly to high temperature stress. The red tube-footed sea urchin may in fact be more sensitive to high temperatures than other sea urchins and respond faster to heat stress. Red tube-footed sea urchins and “DEG-DE miRNA” pairs involved in protein processing of the endoplasmic reticulum may be considered as germplasm resources and molecular markers for future selective breeding.

## Data Availability

The datasets presented in this study can be found in online repositories. The names of the repository/repositories and accession number(s) can be found in the article/Supplementary Material.

## Abbreviations

miRNA, MicroRNA; DEGs, different expression genes; DE miRNA, different expression microRNA; GO, gene ontology; BP, biological processes; MF, molecular functions; CC, cellular components; KEGG, kyoto encyclopedia of genes and genomes; SOD, superoxide dismutase; CAT, catalase; POD, peroxidase; GSH-Px, glutathione peroxidase; qRT-PCR, quantitative real-time PCR.

## References

[B1] Bai X. Q. Pang Z. G. Zhang W. J. Chang Y. Q. Gao Y. X. Ding J. (2015). Relative Expression of Genes Hsp70 and Hsp90 in Sea Urchin strongylocentrotus Intermedius in Thermal Stress. Oceanol. Limnologia Sinica 46, 1034–1039. in Chinese.

[B2] Balakirev E. S. Anisimova M. Pavlyuchkov V. A. Ayala F. J. (2016). DNA Polymorphism and Selection at the Bindin Locus in Three Strongylocentrotus Sp. (Echinoidea). BMC Genet. 17, 66. 10.1186/s12863-016-0374-5 27176219 PMC4866015

[B3] Balakirev E. S. Pavlyuchkov V. A. Ayala F. J. (2008). DNA Variation and Symbiotic Associations in Phenotypically Diverse Sea Urchin Strongylocentrotus Intermedius. Proc. Natl. Acad. Sci. U.S.A. 105, 16218–16223. 10.1073/pnas.0807860105 18852450 PMC2571021

[B4] Bartel D. P. (2004). MicroRNAs: Genomics, Biogenesis, Mechanism, and Function. Cell 116, 281–297. 10.1016/s0092-8674(04)00045-5 14744438

[B5] Blencowe B. J. (2006). Alternative Splicing: New Insights from Global Analyses. Cell 126, 37–47. 10.1016/j.cell.2006.06.023 16839875

[B6] Brown B. E. Dunne R. P. Somerfield P. J. Edwards A. J. Simons W. J. F. Phongsuwan N. (2019). Long-term Impacts of Rising Sea Temperature and Sea Level on Shallow Water Coral Communities over a ∼40 Year Period. Sci. Rep. 9, 8826. 10.1038/s41598-019-45188-x 31217535 PMC6584745

[B7] Chan W. Y. Peplow L. M. Van Oppen M. J. H. (2019). Interspecific Gamete Compatibility and Hybrid Larval Fitness in Reef-Building Corals: Implications for Coral Reef Restoration. Sci. Rep. 9, 4757. 10.1038/s41598-019-41190-5 30894593 PMC6426996

[B8] Chang Y. Q. (2004). Biological Research and Breeding of Sea Cucumber and Sea Urchin. Beijing: China Ocean Press.

[B9] Chang Y. Tian X. Zhang W. Han F. Chen S. Zhou M. (2016). Family Growth and Survival Response to Two Simulated Water Temperature Environments in the Sea Urchin Strongylocentrotus Intermedius. Int. J. Mol. Sci. 17, 1356. 10.3390/ijms17091356 PMC5037655 27589722

[B10] Conaco C. Otto S. Han J.-J. Mandel G. (2006). Reciprocal Actions of REST and a microRNA Promote Neuronal Identity. Proc. Natl. Acad. Sci. U.S.A. 103, 2422–2427. 10.1073/pnas.0511041103 16461918 PMC1413753

[B11] Ding J. Yang D. Chang Y. Wang Y. Zhang W. Chen T. (2017). Comparative Transcriptome Analysis of Tube Feet of Different Colors in the Sea Urchin Strongylocentrotus Intermedius. Genes Genom 39, 1215–1225. 10.1007/s13258-017-0565-0

[B12] Ding W. J. Ding J. Zhang W. J. Ding Y. L. He P. (2015). A Preliminary Study of Rising Temperature on Immune-Related Enzyme and MDA Content in Family of Sea Urchins (Strongylocentrotus Intermedius) with Different Tube Feet Colors. J. Agric. 5, 110–116. in Chinese.

[B13] Ding Y. L. (2014). Family Construction and Preliminary Studies of Sea Urchins (Strongylocentrotus Intermedius) with Different Color Tube Feet and Highly Unsaturated Fatty Acid. Dalian, China: Dalian Ocean University.

[B14] Dong Y. Han G. Ganmanee M. Wang J. (2015). Latitudinal Variability of Physiological Responses to Heat Stress of the Intertidal Limpet Cellana Toreuma along the Asian Coast. Mar. Ecol. Prog. Ser. 529, 107–119. 10.3354/meps11303

[B15] Eggleton P. Bremer E. Dudek E. Michalak M. (2016). Calreticulin, a Therapeutic Target? Expert Opin. Ther. Targets 20, 1137–1147. 10.1517/14728222.2016.1164695 26959400

[B16] Foley N. H. Bray I. M. Tivnan A. Bryan K. Murphy D. M. Buckley P. G. (2010). MicroRNA-184 Inhibits Neuroblastoma Cell Survival through Targeting the Serine/threonine Kinase AKT2. Mol. Cancer 9, 83. 10.1186/1476-4598-9-83 20409325 PMC2864218

[B17] Forgati M. Kandalski P. K. Herrerias T. Zaleski T. Machado C. Souza M. R. D. P. (2017). Effects of Heat Stress on the Renal and Branchial Carbohydrate Metabolism and Antioxidant System of Antarctic Fish. J. Comp. Physiol. B 187, 1137–1154. 10.1007/s00360-017-1088-3 28391590

[B18] Fu L. Gao T. Jiang H. Zhang Y. Pan J. (2021). Integrated miRNA-mRNA Transcriptomic Analysis of Hepatopancreas Reveals Molecular Strategies in Chinese Mitten Crab (Eriocheir Sinensis) under Acute Nitrite Stress. Aquacult Int. 29, 1015–1030. 10.1007/s10499-021-00672-y

[B19] García-Echauri L. L. Liggins G. Cetina-Heredia P. Roughan M. Coleman M. A. Jeffs A. (2020). Future Ocean Temperature Impacting the Survival Prospects of Post-larval Spiny Lobsters. Mar. Environ. Res. 156, 104918. 10.1016/j.marenvres.2020.104918 32174338

[B20] Gonzalez M. Font A. Gallego A. (2017). Antioxidants and Chaperones Gene Expression in Immune Cells of Antarctic Sea Urchin by Stress Inductors. SCAR Biol. 7, 10–14. 10.13140/RG.2.2.19886.20804

[B21] Gotoh T. Terada K. Oyadomari S. Mori M. (2004). hsp70-DnaJ Chaperone Pair Prevents Nitric Oxide- and CHOP-Induced Apoptosis by Inhibiting Translocation of Bax to Mitochondria. Cell Death Differ. 11, 390–402. 10.1038/sj.cdd.4401369 14752510

[B22] Gouda H. Agatsuma Y. (2020). Effect of High Temperature on Gametogenesis of the Sea Urchin Strongylocentrotus Intermedius in the Sea of Japan, Northern Hokkaido, Japan. J. Exp. Mar. Biol. Ecol. 525, 151324. 10.1016/j.jembe.2020.151324

[B23] Grabherr M. G. Haas B. J. Yassour M. Levin J. Z. Thompson D. A. Amit I. (2011). Full-length Transcriptome Assembly from RNA-Seq Data without a Reference Genome. Nat. Biotechnol. 29, 644–652. 10.1038/nbt.1883 21572440 PMC3571712

[B24] Grundy J. E. Storey K. B. (1998). Antioxidant Defenses and Lipid Peroxidation Damage in Estivating Toads, *Scaphiopus couchii* . J. Comp. Physiology B Biochem. Syst. Environ. Physiology 168, 132–142. 10.1007/s003600050129 9542148

[B25] Han L. Ding J. Wang H. Zuo R. Quan Z. Fan Z. (2019). Molecular Characterization and Expression of SiFad1 in the Sea Urchin (Strongylocentrotus Intermedius). Gene 705, 133–141. 10.1016/j.gene.2019.04.043 31004713

[B26] Harvell C. D. Mitchell C. E. Ward J. R. Altizer S. Dobson A. P. Ostfeld R. S. (2002). Climate Warming and Disease Risks for Terrestrial and Marine Biota. Science 296, 2158–2162. 10.1126/science.1063699 12077394

[B27] Jiang Y. Zhang S. Xu J. Feng J. Mahboob S. Al-Ghanim K. A. (2014). Comparative Transcriptome Analysis Reveals the Genetic Basis of Skin Color Variation in Common Carp. PLoS One 9, e108200. 10.1371/journal.pone.0108200 25255374 PMC4177847

[B28] Kaluza D. Kroll J. Gesierich S. Manavski Y. Boeckel J.-N. Doebele C. (2013). Histone Deacetylase 9 Promotes Angiogenesis by Targeting the Antiangiogenic microRNA-17-92 Cluster in Endothelial Cells. Arterioscler. Thromb. Vasc. Biol. 33, 533–543. 10.1161/atvbaha.112.300415 23288173

[B29] Lan C. Chen Q. Li J. (2016). Grouping miRNAs of Similar Functions via Weighted Information Content of Gene Ontology. BMC Bioinforma. 17, 507. 10.1186/s12859-016-1367-0 PMC5260111 28155659

[B30] Lee A. S. (2005). The ER Chaperone and Signaling Regulator GRP78/BiP as a Monitor of Endoplasmic Reticulum Stress. Methods 35, 373–381. 10.1016/j.ymeth.2004.10.010 15804610

[B31] Li C. Fang H. Xu D. (2019). Effect of Seasonal High Temperature on the Immune Response in Apostichopus Japonicus by Transcriptome Analysis. Fish Shellfish Immunol. 92, 765–771. 10.1016/j.fsi.2019.07.012 31288099

[B32] Li C. Xu D. (2018). Understanding microRNAs Regulation in Heat Shock Response in the Sea Cucumber Apostichopus Japonicus. Fish Shellfish Immunol. 81, 214–220. 10.1016/j.fsi.2018.07.034 30016683

[B33] Li C. Zhao W. Qin C. Yu G. Ma Z. Guo Y. (2021). Comparative Transcriptome Analysis Reveals Changes in Gene Expression in Sea Cucumber (Holothuria Leucospilota) in Response to Acute Temperature Stress. Comp. Biochem. Physiology Part D Genomics Proteomics 40, 100883. 10.1016/j.cbd.2021.100883 34303260

[B34] Li J. Zhang Y. Mao F. Tong Y. Liu Y. Zhang Y. (2017). Characterization and Identification of Differentially Expressed Genes Involved in Thermal Adaptation of the Hong Kong Oyster Crassostrea Hongkongensis by Digital Gene Expression Profiling. Front. Mar. Sci. 4, 112. 10.3389/fmars.2017.00112

[B35] Liu C. Teng Z.-Q. Santistevan N. J. Szulwach K. E. Guo W. Jin P. (2010). Epigenetic Regulation of miR-184 by MBD1 Governs Neural Stem Cell Proliferation and Differentiation. Cell Stem Cell 6, 433–444. 10.1016/j.stem.2010.02.017 20452318 PMC2867837

[B36] Livak K. J. Schmittgen T. D. (2001). Analysis of Relative Gene Expression Data Using Real-Time Quantitative PCR and the 2−ΔΔCT Method. Methods 25, 402–408. 10.1006/meth.2001.1262 11846609

[B37] Lu Y. Wu Z. Song Z. Xiao P. Liu Y. Zhang P. (2016). Insight into the Heat Resistance of Fish via Blood: Effects of Heat Stress on Metabolism, Oxidative Stress and Antioxidant Response of Olive Flounder *Paralichthys olivaceus* and Turbot *Scophthalmus maximus* . Fish Shellfish Immunol. 58, 125–135. 10.1016/j.fsi.2016.09.008 27633671

[B38] Luo W. Xu Y. Liu X. J. Wang C. F. (2017). Effect of Water Temperature on Serum Content of Reactive Oxygen Species and Antioxidant Defense System in Grass Carp Ctenopharyngodon Idellusin. Freshw. Fish. 47, 3–11. 10.13721/j.cnki.dsyy.2017.04.001

[B39] Lv M. Chen H. Shao Y. Li C. Zhang W. Zhao X. (2017). miR-92a Regulates Coelomocytes Apoptosis in Sea Cucumber Apostichopus Japonicus via Targeting Aj14-3-3 ζ *In Vivo* . Fish Shellfish Immunol. 69, 211–217. 10.1016/j.fsi.2017.08.033 28860073

[B40] Ma C.-y. Zhu X.-l. Liao M.-l. Dong S.-l. Dong Y.-w. (2021). Heat Sensitivity of Mariculture Species in China. ICES J. Mar. Sci. 78, 2922–2930. 10.1093/icesjms/fsab168

[B41] Ma F. Liu Z. Huang J. Li Y. Kang Y. Liu X. (2019). High-throughput Sequencing Reveals microRNAs in Response to Heat Stress in the Head Kidney of Rainbow Trout (*Oncorhynchus mykiss*). Funct. Integr. Genomics 19, 775–786. 10.1007/s10142-019-00682-3 31076931

[B42] Makeyev E. V. Zhang J. Carrasco M. A. Maniatis T. (2007). The MicroRNA miR-124 Promotes Neuronal Differentiation by Triggering Brain-specific Alternative Pre-mRNA Splicing. Mol. Cell 27, 435–448. 10.1016/j.molcel.2007.07.015 17679093 PMC3139456

[B43] Manni I. Artuso S. Careccia S. Giulia Rizzo M. Baserga R. Piaggio G. (2009). The microRNA miR‐92 Increases Proliferation of Myeloid Cells and by Targeting P63 Modulates the Abundance of its Isoforms. FASEB J. 23, 3957–3966. 10.1096/fj.09-131847 19608627

[B44] Mao X. Cai T. Olyarchuk J. G. Wei L. (2005). Automated Genome Annotation and Pathway Identification Using the KEGG Orthology (KO) as a Controlled Vocabulary. Bioinformatics 21, 3787–3793. 10.1093/bioinformatics/bti430 15817693

[B45] Martin M. (2011). CUTADAPT Removes Adapter Sequences from High-Throughput Sequencing Reads. EMBnet J. 17, 10–12. 10.14806/ej.17.1.200

[B46] Morimoto R. I. Sarge K. D. Abravaya K. (1992). Transcriptional Regulation of Heat Shock Genes. A Paradigm for Inducible Genomic Responses. J. Biol. Chem. 267, 21987–21990. 10.1016/s0021-9258(18)41621-3 1429548

[B47] Nigam P. A. (2014). MicroRNAs Regulate Sea Urchin Gut Specification and Development by Targeting Components of the Wnt Signaling Pathway. Newark, America: University of Delaware, 1562407.

[B48] Olive V. Bennett M. J. Walker J. C. Ma C. Jiang I. Cordon-Cardo C. (2009). miR-19 Is a Key Oncogenic Component of Mir-17-92. Genes Dev. 23, 2839–2849. 10.1101/gad.1861409 20008935 PMC2800084

[B49] Olive V. Jiang I. He L. (2010). mir-17-92, a Cluster of miRNAs in the Midst of the Cancer Network. Int. J. Biochem. Cell Biol. 42, 1348–1354. 10.1016/j.biocel.2010.03.004 20227518 PMC3681296

[B50] Pan Y. Zhao Z. Zhou Z. (2021). Identification of miRNAs in Sea Urchin *Strongylocentrotus purpuratus* Larvae Response to pH Stress. Aquac. Res. 52, 4735–4744. 10.1111/are.15307

[B51] Paredes E. (2016). Biobanking of a Marine Invertebrate Model Organism: The Sea Urchin. J. Mar. Sci. Eng. 4 (1), 7. 10.3390/jmse4010007

[B52] Park J. K. Peng H. Yang W. Katsnelson J. Volpert O. Lavker R. M. (2017). miR‐184 Exhibits Angiostatic Properties via Regulation of Akt and VEGF Signaling Pathways. FASEB J. 31, 256–265. 10.1096/fj.201600746R 27825105 PMC5161520

[B53] Qiang J. Bao W. J. Tao F. Y. He J. Li X. H. Xu P. (2017). The Expression Profiles of miRNA-mRNA of Early Response in Genetically Improved Farmed tilapia (*Oreochromis niloticus*) Liver by Acute Heat Stress. Sci. Rep. 7, 8705. 10.1038/s41598-017-09264-4 28821885 PMC5562739

[B54] Samanta M. P. Tongprasit W. Istrail S. Cameron R. A. Tu Q. Davidson E. H. (2006). The Transcriptome of the Sea Urchin Embryo. Science 314, 960–962. 10.1126/science.1131898 17095694

[B55] Sanford E. Kelly M. W. (2011). Local Adaptation in Marine Invertebrates. Annu. Rev. Mar. Sci. 3, 509–535. 10.1146/annurev-marine-120709-142756 21329215

[B56] Schröder M. Kaufman R. J. (2005). The Mammalian Unfolded Protein Response. Annu. Rev. Biochem. 74, 739–789. 10.1146/annurev.biochem.73.011303.074134 15952902

[B57] Sha Z.-X. Liu H. Wang Q.-L. Liu Y. Lu Y. Li M. (2012). Channel Catfish (*Ictalurus punctatus*) Protein Disulphide Isomerase, PDIA6: Molecular Characterization and Expression Regulated by Bacteria and Virus Inoculation. Fish Shellfish Immunol. 33, 220–228. 10.1016/j.fsi.2012.04.014 22561356

[B58] Shi D. Zhao C. Chen Y. Ding J. Zhang L. Chang Y. (2020). Transcriptomes Shed Light on Transgenerational and Developmental Effects of Ocean Warming on Embryos of the Sea Urchin Strongylocentrotus Intermedius. Sci. Rep. 10, 7931. 10.1038/s41598-020-64872-x 32404890 PMC7221070

[B59] Siikavuopio S. I. James P. Lysne H. Sæther B. S. Samuelsen T. A. Mortensen A. (2012). Effects of Size and Temperature on Growth and Feed Conversion of Juvenile Green Sea Urchin (*Strongylocentrotus droebachiensis*). Aquaculture 354-355, 27–30. 10.1016/j.aquaculture.2012.04.036

[B60] Somero G. N. (2012). The Physiology of Global Change: Linking Patterns to Mechanisms. Annu. Rev. Mar. Sci. 4, 39–61. 10.1146/annurev-marine-120710-100935 22457968

[B61] Stepicheva N. A. Song J. L. (2015). microRNA-31 Modulates Skeletal Patterning in the Sea Urchin Embryos. Development 142, 3769–3780. 10.1242/dev.127969 26400092 PMC4647217

[B62] Strøm J. F. Thorstad E. B. Rikardsen A. H. (2020). Thermal Habitat of Adult Atlantic Salmon *Salmo salar* in a Warming Ocean. J. Fish. Biol. 96, 327–336. 10.1111/jfb.14187 31661157

[B63] Takahashi Y. Chen Q. Rajala R. V. S. Ma J.-x. (2015). MicroRNA-184 Modulates Canonical Wnt Signaling through the Regulation of Frizzled-7 Expression in the Retina with Ischemia-Induced Neovascularization. FEBS Lett. 589, 1143–1149. 10.1016/j.febslet.2015.03.010 25796186 PMC4406844

[B64] Tamatani M. Matsuyama T. Yamaguchi A. Mitsuda N. Tsukamoto Y. Taniguchi M. (2001). ORP150 Protects against Hypoxia/ischemia-Induced Neuronal Death. Nat. Med. 7, 317–323. 10.1038/85463 11231630

[B65] Tan W. Li Y. Lim S. G. Tan T. M. (2014). miR-106b-25/miR-17-92clusters: Polycistrons with Oncogenic Roles in Hepatocellular Carcinoma. World J. Gastroenterol. 20, 5962–5972. 10.3748/wjg.v20.i20.5962 24876719 PMC4033436

[B66] Tsuchida A. Ohno S. Wu W. Borjigin N. Fujita K. Aoki T. (2011). miR-92 Is a Key Oncogenic Component of the miR-17-92 Cluster in Colon Cancer. Cancer Sci. 102 **,** 2264–2271. 10.1111/j.1349-7006.2011.02081.x 21883694

[B67] Wang H. Ding J. Ding S. Chang Y. (2019). Transcriptome Analysis to Characterize the Genes Related to Gonad Growth and Fatty Acid Metabolism in the Sea Urchin Strongylocentrotus Intermedius. Genes Genom 41, 1397–1415. 10.1007/s13258-019-00864-0 31485990

[B68] Wang Z. Feng Y. Li J. Zou J. Fan L. (2020). Integrative microRNA and mRNA Analysis Reveals Regulation of ER Stress in the Pacific White Shrimp Litopenaeus Vannamei under Acute Cold Stress. Comp. Biochem. Physiology Part D Genomics Proteomics 33, 100645. 10.1016/j.cbd.2019.100645 31794884

[B69] Winston G. W. (1991). Oxidants and Antioxidants in Aquatic Animals. Comp. Biochem. Physiology Part C Comp. Pharmacol. 100, 173–176. 10.1016/0742-8413(91)90148-m 1677850

[B70] Yang C. Gao Q. Liu C. Wang L. Zhou Z. Gong C. (2017). The Transcriptional Response of the Pacific Oyster *Crassostrea gigas* against Acute Heat Stress. Fish Shellfish Immunol. 68 **,** 132–143. 10.1016/j.fsi.2017.07.016 28698121

[B71] Yang R. Zheng T. Cai X. Yu Y. Yu C. Guo L. (2013). Genome-wide Analyses of Amphioxus microRNAs Reveal an Immune Regulation via miR-92d Targeting C3. J. Immunol. 190 **,** 1491–1500. 10.4049/jimmunol.1200801 23335747

[B72] Yasuhara M. Danovaro R. (2016). Temperature Impacts on Deep-Sea Biodiversity. Biol. Rev. 91, 275–287. 10.1111/brv.12169 25523624

[B73] Young M. D. Wakefield M. J. Smyth G. K. Oshlack A. (2010). Gene Ontology Analysis for RNA-Seq: Accounting for Selection Bias. Genome Biol. 11, R14. 10.1186/gb-2010-11-2-r14 20132535 PMC2872874

[B74] Zeng G. E. Lian S. M. Cheng X. H. Hua Z. L. Quan Q. Y. (2006). EOF Analysis of Intra-seasonal Variabilities of SST in the East China Sea and Yellow Sea. Adv. Mar. Sci. 24, 147–155. 10.1016/S1001-8042(06)60021-3

[B75] Zhan Y. Li Y. Cui D. Pei Q. Sun J. Zhang W. (2018). Identification and Characterization of microRNAs from the Tube Foot in the Sea Urchin Strongylocentrotus Intermedius. Heliyon 4, e00668. 10.1016/j.heliyon.2018.e00668 30003162 PMC6039759

[B76] Zhang W. Zhao C. Liu P. Chang Y. (2010). First Report on Tube Feet Differential Pigmentation in the Cultivated Sea Urchin Strongylocentrotus Intermedius (Agassiz, 1863) and its Relationship with Growth Performance. Aquac. Res. 41, e706–e708. 10.1111/j.1365-2109.2010.02521.x

[B77] Zhao C. Zhang L. Shi D. Ding J. Yin D. Sun J. (2018). Transgenerational Effects of Ocean Warming on the Sea Urchin Strongylocentrotus Intermedius. Ecotoxicol. Environ. Saf. 151, 212–219. 10.1016/j.ecoenv.2018.01.014 29353170

[B78] Zhao X. Y. Chang Y. Q. Ding J. Zhang W. J. (2011). Immune Effect of Four Immune Opotentiators on Two Sea Urchin (*Strongylocentrotus Intermedius*) with Different Tube Foot Colours. Adv. Mar. Sci. 29, 8. in Chinese.

[B79] Zhao Z. W. Zhang M. Chen L. Y. Gong D. Xia X. D. Yu X. H. (2018). Heat Shock Protein 70 Accelerates Atherosclerosis by Downregulating the Expression of ABCA1 and ABCG1 through the JNK/Elk-1 Pathway. Biochim. Biophys. Acta Mol. Cell Biol. Lipids 1863, 806–822. 10.1016/j.bbalip.2018.04.011 29678642

[B80] Zheng J. Cao J. Mao Y. Su Y. Wang J. (2018). Identification of microRNAs with Heat Stress Responsive and Immune Properties in *Marsupenaeus japonicus* Based on Next-Generation Sequencing and Bioinformatics Analysis: Essential Regulators in the Heat Stress-Host Interactions. Fish. Shellfish Immunol. 81, 390–398. 10.1016/j.fsi.2018.05.030 29778844

[B81] Zhou C. Q. Zhou P. Ren Y. L. Cao L. H. Wang J. L. (2019). Physiological Response and miRNA-mRNA Interaction Analysis in the Head Kidney of Rainbow Trout Exposed to Acute Heat Stress. J. Therm. Biol. 83, 134–141. 10.1016/j.jtherbio.2019.05.014 31331511

[B82] Zhou J. Schmid T. Frank R. Brüne B. (2004). PI3K/Akt Is Required for Heat Shock Proteins to Protect Hypoxia-Inducible Factor 1alpha from pVHL-independent Degradation. J. Biol. Chem. 279, 13506–13513. 10.1074/jbc.M310164200 14726529

