# Supplementary material for: Expression Regulation Mechanisms of Sea Urchin (Strongylocentrotus intermedius) Under the High Temperature: New Evidence for the miRNA-mRNA Interaction Involvement
Source: Front Genet. 2022 Jun 29;13:876308. doi: 10.3389/fgene.2022.876308 (PMC9277089; doi:10.3389/fgene.2022.876308)
Supplement: Supplementary file 1 [file Table1.DOCX]

Supplementary Table 1 Primers used for RNA-seq verification

| Category | Name | Primer sequences (5′→3′) |
| --- | --- | --- |
| mRNA | HSP70 | F: CACTGATACCGAGCGTCTGATTGG  R: TCACACATGCGTCTCCGAAGTTTC |
|  | DnaJ11 | F: TGGTAATGGCGGACAGAGGAGAG  R: CCAGGTGCTTCCGTTGCTACAG |
|  | PDIA4 | F: CCCTCAACTGGTGGCTTTCTTCTC  R: CCTTGGCTACCTCAACGATCTTCTG |
|  | PDIA6 | F: GTGCCAACAAGAAGAGCCCTACAG  R: CCTCCTCCTCCTCCTCCTCCTC |
|  | GRP78 | F: TCAGCGGAGAGGCAGAGACAAG  R: GGTACGACGGTGTTCCTTCCAATG |
|  | HYOU1 | F: CTGGTGGTTCTAACGCCGATGATG  R: GTTCTGCTTCTGCTTCTCCTTCTCC |
|  | TUBA | F: GGAGGATGCTGCCAACAACTACG  R: CCGAAGCTGTGGAAGATGAGGAATC |
|  | TUBB | F: ACCTTCATCGGCAACAGCACTG  R: ACTCCATCTCGTCCATACCCTCAC |
|  | MMR1 | F: TGCTACTACGTCCCTGATGGTCTG  R: CCTCTTGCCGATGTCTGTCTGAAC |
|  | CALR | F: CCTGCTTGTGCTTGATGCTTGTTG  R: ACGGACTCTACCCAGCGAGATTC |
|  | HYAL | F: TTGCGAAGGCTCATACACTACTTGG  R: GCCAGATGTCCACCGTCAGATTC |
|  | GANAB | F:TTGAGCATCAGCGGGAGAGGAG  R: GTCTTCCACGGATCTGTAACCAACC |
|  | SAR1B | F: CGCAGGCAAGACGACGTTACTC  R: CGATCCTCAGTTCTTCCGATGTTGG |
|  | SL-11 | F: AGTGCTCTCGTCTACTCCTTGCC  R: TCCTTGATGGCTTTGAACTCGTCTG |
|  | MGST-1 | F: CCATCAGAACGACCTGGAGAACATC  R: GAGGCGACGAAGATACGGTAATGC |
|  | GST3 | F: TGCTCCAGTCCGTTGCTTGAATG  R:TTTGCTCGTCGTCCAGGTTACTTG |
|  | C3p | F: CTACGCTCTCCTGGCACAACTTG  R: TCCTCCACCATAGTTCTGTTGCTTG |
|  | CTL | F: GATGGAACCAGCGTGGACTATTACC  R: ACTTCTGCCTGTCATTGTCACTGC |
|  | CYP2J6 | F: CTGGTCGCCGTGTCTGTATTGG  R: GTGGGTGAGTCGTCAGGTTTCTTG |
|  | CYP3A24 | F: CGGATGGAAACCTGCCCTCAAAC  R: CGCTTGACTGCTCGGAGAGTAATG |
| Reference gene | 18s rRNA | F: GTTCGAAGGCGATCAGATAC  R: CTGTCAATCCTCACTGTGTC |
| miRNA | spu-mir-184-p5 | F: TCCTTATCATTCTCTTGCCCGGC |
|  | spu-mir-92a-p5 | F: TCAGGTCGTGACTCGTGCTCAATATT |
|  | spu-mir-92c-p5 | F: CCTGGTCGTGAGGAGTTGCAATTTG |
|  | sko-miR-184-3p | F: TGGACGGAGAACTGATAAGGGCA |
|  | lva-miR-92b-3p | F: ATATTGCACTCGTCCCGGCC |
|  | pmi-mir-92c-3p | F: ATATTGCACTCGTCCCGGCC |
|  | PC-3p-20672 | F: CGGTTCGAACCCCAGCTGAAT |
|  | spu-mir-124-p5 | F: CGCAGTGTTCACTGTGATCCTTGAT |
|  | PC-3p-60_406134 | F: GCATTGCACTCATCTCGGTCTGA |
|  | Mir-R | R:GCTGTCAACGATACGCTACGTAACGGCATGACAGTG |
| Reference miRNA | U6 | F:ACGCAAATTCGTGAAGCGTT |
