# Supplementary figures and images for: Expression Regulation Mechanisms of Sea Urchin (Strongylocentrotus intermedius) Under the High Temperature: New Evidence for the miRNA-mRNA Interaction Involvement
Source: Front Genet. 2022 Jun 29;13:876308. doi: 10.3389/fgene.2022.876308 (PMC9277089; doi:10.3389/fgene.2022.876308)

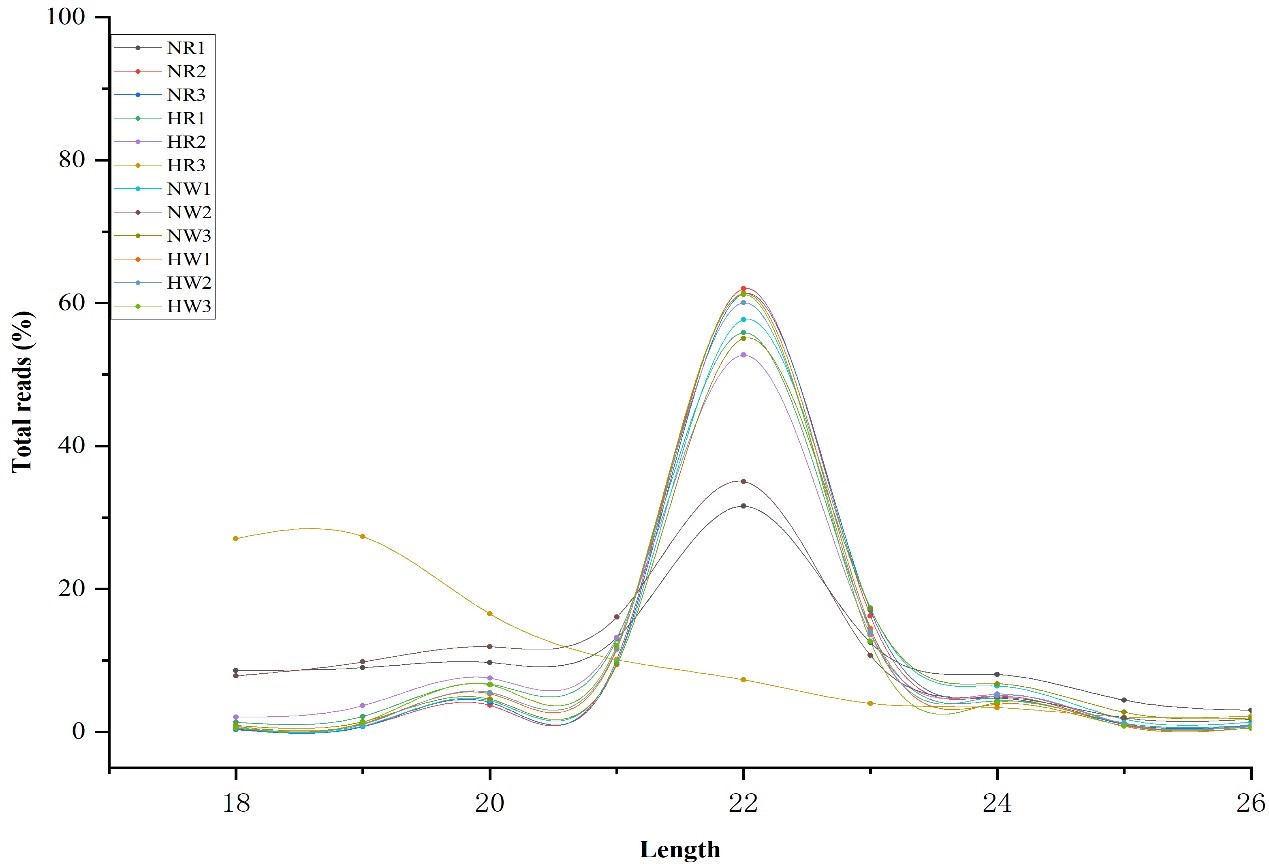

Supplement: Supplementary file 3 [file Image1.JPEG]

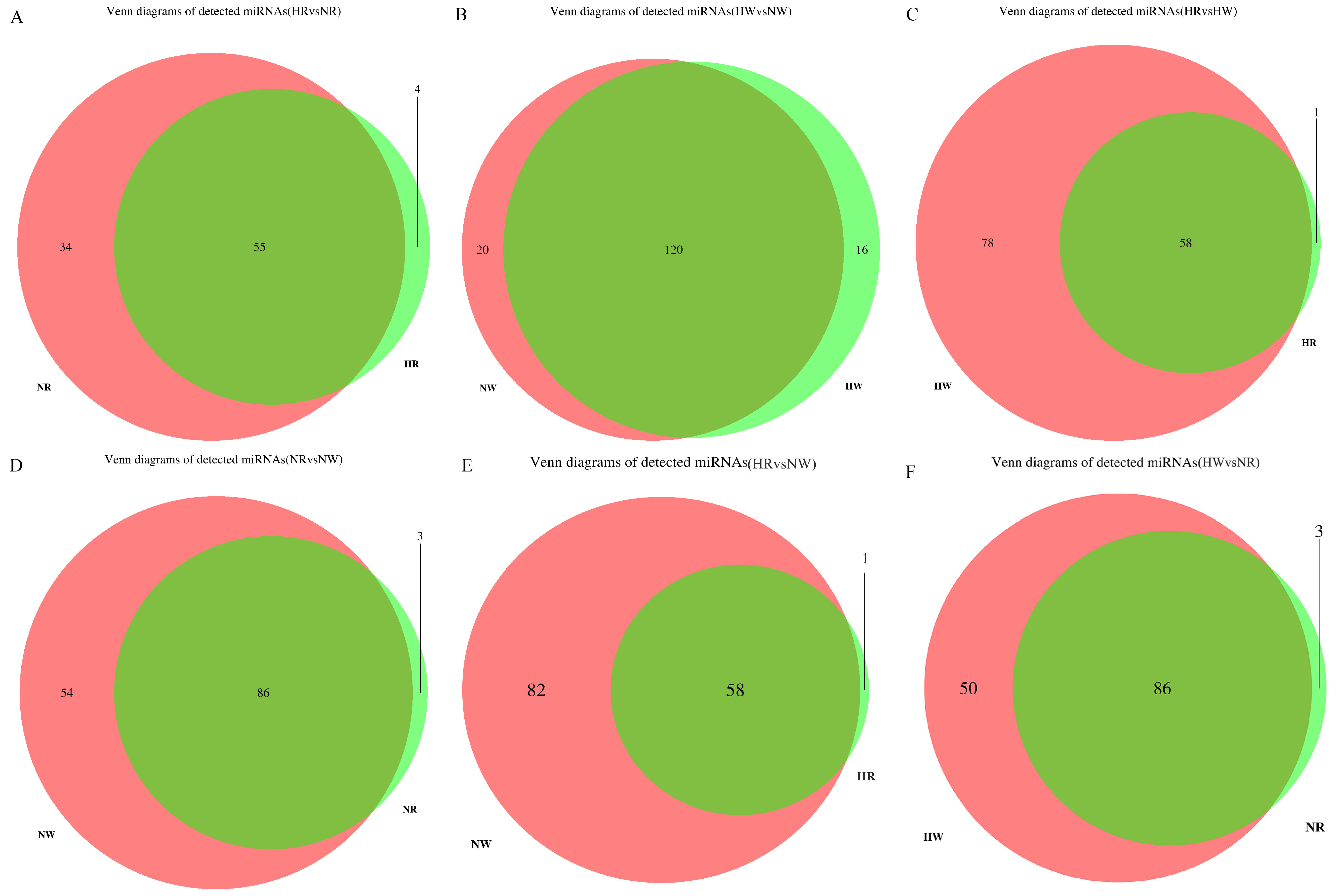

Supplement: Supplementary file 5 [file Image2.JPEG]
