# Supplementary material for: Expression Regulation Mechanisms of Sea Urchin (Strongylocentrotus intermedius) Under the High Temperature: New Evidence for the miRNA-mRNA Interaction Involvement
Source: Front Genet. 2022 Jun 29;13:876308. doi: 10.3389/fgene.2022.876308 (PMC9277089; doi:10.3389/fgene.2022.876308)
Supplement: Supplementary file 4 [file Table7.DOCX]

Supplementary Table 6 Overview of reads from raw data to cleaned sequences (miRNA-Seq)

|  |  | Raw reads | 3ADT&length filter | Junk reads | Rfam | Repeats | valid reads | rRNA | tRNA | snoRNA | snRNA | other Rfam RNA |
| --- | --- | --- | --- | --- | --- | --- | --- | --- | --- | --- | --- | --- |
| NR1 | Total | 11169788 | 8495852 | 10852 | 313130 | 26390 | 2347876 | 289664 | 2659 | 4589 | 7364 | 8854 |
|  | % of Total | 100.00 | 76.06 | 0.10 | 2.80 | 0.24 | 21.02 | 2.59 | 0.02 | 0.04 | 0.07 | 0.08 |
|  | uniq | 541137 | 308434 | 3341 | 2814 | 151 | 226486 | 2074 | 177 | 221 | 122 | 220 |
|  | % of uniq | 100.00 | 57.00 | 0.62 | 0.52 | 0.03 | 41.85 | 0.38 | 0.03 | 0.04 | 0.02 | 0.04 |
| NR2 | Total | 20808944 | 1756692 | 4033 | 86470 | 5133 | 18957951 | 56959 | 19148 | 5103 | 3840 | 1420 |
|  | % of Total | 100.00 | 8.44 | 0.02 | 0.42 | 0.02 | 91.10 | 0.27 | 0.09 | 0.02 | 0.02 | 0.01 |
|  | uniq | 673571 | 434668 | 1674 | 2165 | 180 | 234997 | 1237 | 439 | 258 | 132 | 99 |
|  | % of uniq | 100.00 | 64.53 | 0.25 | 0.32 | 0.03 | 34.89 | 0.18 | 0.07 | 0.04 | 0.02 | 0.01 |
| NR3 | Total | 29531299 | 2245861 | 8047 | 196156 | 8938 | 27075811 | 132871 | 38912 | 14676 | 5793 | 3904 |
|  | % of Total | 100.00 | 7.61 | 0.03 | 0.66 | 0.03 | 91.69 | 0.45 | 0.13 | 0.05 | 0.02 | 0.01 |
|  | uniq | 991088 | 560245 | 3598 | 5306 | 251 | 421860 | 3140 | 1204 | 434 | 224 | 304 |
|  | % of uniq | 100.00 | 56.53 | 0.36 | 0.54 | 0.03 | 42.57 | 0.32 | 0.12 | 0.04 | 0.02 | 0.03 |
| HR1 | Total | 12128673 | 2039564 | 4079 | 145804 | 6663 | 9935944 | 109329 | 21409 | 4835 | 2948 | 7283 |
|  | % of Total | 100.00 | 16.82 | 0.03 | 1.20 | 0.05 | 81.92 | 0.90 | 0.18 | 0.04 | 0.02 | 0.06 |
|  | uniq | 1040446 | 711318 | 2015 | 4631 | 211 | 322414 | 3014 | 720 | 255 | 137 | 505 |
|  | % of uniq | 100.00 | 68.37 | 0.19 | 0.45 | 0.02 | 30.99 | 0.29 | 0.07 | 0.02 | 0.01 | 0.05 |
| HR2 | Total | 15445365 | 4358691 | 5531 | 214798 | 14082 | 10860865 | 147343 | 38187 | 11690 | 3786 | 13792 |
|  | % of Total | 100.00 | 28.22 | 0.04 | 1.39 | 0.09 | 70.32 | 0.95 | 0.25 | 0.08 | 0.02 | 0.09 |
|  | uniq | 1289363 | 821812 | 2190 | 9204 | 317 | 456059 | 5800 | 1956 | 343 | 154 | 951 |
|  | % of uniq | 100.00 | 63.74 | 0.17 | 0.71 | 0.02 | 35.37 | 0.45 | 0.15 | 0.03 | 0.01 | 0.07 |
| HR3 | Total | 13378897 | 11903191 | 4132 | 313251 | 3983 | 1157842 | 292814 | 5091 | 3317 | 2740 | 9289 |
|  | % of Total | 100.00 | 88.97 | 0.03 | 2.34 | 0.03 | 8.65 | 2.19 | 0.04 | 0.02 | 0.02 | 0.07 |
|  | uniq | 447162 | 319075 | 1066 | 2509 | 62 | 124484 | 1814 | 392 | 32 | 29 | 242 |
|  | % of uniq | 100.00 | 71.36 | 0.24 | 0.56 | 0.01 | 27.84 | 0.41 | 0.09 | 0.01 | 0.01 | 0.05 |
| NW1 | Total | 18404475 | 2374030 | 5187 | 536624 | 7074 | 15484017 | 80939 | 434620 | 9238 | 5089 | 6738 |
|  | % of Total | 100.00 | 12.90 | 0.03 | 2.92 | 0.04 | 84.13 | 0.44 | 2.36 | 0.05 | 0.03 | 0.04 |
|  | uniq | 889651 | 550260 | 2601 | 6197 | 246 | 330512 | 2491 | 2804 | 459 | 144 | 299 |
|  | % of uniq | 100.00 | 61.85 | 0.29 | 0.70 | 0.03 | 37.15 | 0.28 | 0.32 | 0.05 | 0.02 | 0.03 |
| NW2 | Total | 17211754 | 10098609 | 7874 | 582324 | 43642 | 6505951 | 396037 | 122918 | 17608 | 7513 | 38248 |
|  | % of Total | 100.00 | 58.67 | 0.05 | 3.38 | 0.25 | 37.80 | 2.30 | 0.71 | 0.10 | 0.04 | 0.22 |
|  | uniq | 2627443 | 1900242 | 3928 | 18130 | 598 | 704950 | 10209 | 5155 | 483 | 445 | 1838 |
|  | % of uniq | 100.00 | 72.32 | 0.15 | 0.69 | 0.02 | 26.83 | 0.39 | 0.20 | 0.02 | 0.02 | 0.07 |
| NW3 | Total | 21991018 | 3426295 | 12645 | 1998008 | 10618 | 16551191 | 253557 | 1710097 | 8378 | 3959 | 22017 |
|  | % of Total | 100.00 | 15.58 | 0.06 | 9.09 | 0.05 | 75.26 | 1.15 | 7.78 | 0.04 | 0.02 | 0.10 |
|  | uniq | 1202042 | 664023 | 4824 | 15007 | 253 | 518118 | 7327 | 6098 | 316 | 185 | 1081 |
|  | % of uniq | 18404475 | 2374030 | 5187 | 536624 | 0.02 | 43.10 | 0.61 | 0.51 | 0.03 | 0.02 | 0.09 |
| HW1 | Total | 13039823 | 1039393 | 2375 | 128953 | 5519 | 11864654 | 62572 | 55077 | 6952 | 2889 | 1463 |
|  | % of Total | 100.00 | 7.97 | 0.02 | 0.99 | 0.04 | 90.99 | 0.48 | 0.42 | 0.05 | 0.02 | 0.01 |
|  | uniq | 548854 | 346628 | 1301 | 2502 | 169 | 198347 | 1351 | 626 | 293 | 111 | 121 |
|  | % of uniq | 100.00 | 63.15 | 0.24 | 0.46 | 0.03 | 36.14 | 0.25 | 0.11 | 0.05 | 0.02 | 0.02 |
| HW2 | Total | 11299413 | 793238 | 2640 | 127774 | 3366 | 10373463 | 40702 | 78723 | 4965 | 1683 | 1701 |
|  | % of Total | 100.00 | 7.02 | 0.02 | 1.13 | 0.03 | 91.81 | 0.36 | 0.70 | 0.04 | 0.01 | 0.02 |
|  | uniq | 507710 | 279832 | 1495 | 2908 | 151 | 223422 | 1666 | 756 | 244 | 87 | 155 |
|  | % of uniq | 100.00 | 55.12 | 0.29 | 0.57 | 0.03 | 44.01 | 0.33 | 0.15 | 0.05 | 0.02 | 0.03 |
| HW3 | Total | 16753466 | 1272385 | 4362 | 76157 | 5330 | 15396726 | 57818 | 5441 | 7733 | 3005 | 2160 |
|  | % of Total | 100.00 | 7.59 | 0.03 | 0.45 | 0.03 | 91.90 | 0.35 | 0.03 | 0.05 | 0.02 | 0.01 |
|  | uniq | 586661 | 362094 | 1955 | 2165 | 181 | 220379 | 1257 | 323 | 308 | 119 | 158 |
|  | % of uniq | 100.00 | 61.72 | 0.33 | 0.37 | 0.03 | 37.56 | 0.21 | 0.06 | 0.05 | 0.02 | 0.03 |
