# Supplementary material for: Expression Regulation Mechanisms of Sea Urchin (Strongylocentrotus intermedius) Under the High Temperature: New Evidence for the miRNA-mRNA Interaction Involvement
Source: Front Genet. 2022 Jun 29;13:876308. doi: 10.3389/fgene.2022.876308 (PMC9277089; doi:10.3389/fgene.2022.876308)
Supplement: Supplementary file 7 [file Table2.DOCX]

Supplementary Table 2 Sequencing data quality processing results

| Sample | Raw reads | Raw base | Clean reads | Clean base | Clean Ratio  (reads) | Q20 | Q30 | GC% |
| --- | --- | --- | --- | --- | --- | --- | --- | --- |
| HR1 | 41327762 | 6.20G | 40255610 | 5.62G | 97.41 | 97.89 | 93.46 | 42.22 |
| HR2 | 56834206 | 8.53G | 48242788 | 6.74G | 84.88 | 98.40 | 94.89 | 45.86 |
| HR3 | 50142040 | 7.52G | 45648034 | 6.38G | 91.04 | 98.22 | 94.46 | 45.74 |
| HW1 | 47671512 | 7.15G | 46498588 | 6.49G | 97.54 | 97.86 | 93.42 | 42.70 |
| HW2 | 44914914 | 6.74G | 43881072 | 6.12G | 97.70 | 97.84 | 93.35 | 42.31 |
| HW3 | 49514910 | 7.43G | 48065808 | 6.71G | 97.07 | 97.98 | 93.69 | 44.66 |
| NR1 | 51786228 | 7.77G | 49711912 | 6.94G | 95.99 | 98.13 | 94.18 | 43.82 |
| NR2 | 38812224 | 5.82G | 37812016 | 5.27G | 97.42 | 97.86 | 93.44 | 42.52 |
| NR3 | 47683042 | 7.15G | 46463046 | 6.49G | 97.44 | 97.97 | 93.73 | 44.79 |
| NW1 | 42313844 | 6.35G | 41149336 | 5.74G | 97.25 | 97.92 | 93.61 | 43.26 |
| NW2 | 40872664 | 6.13G | 32804248 | 4.58G | 80.26 | 98.12 | 94.21 | 45.95 |
| NW3 | 44328548 | 6.65G | 43252056 | 6.04G | 97.57 | 97.97 | 93.67 | 43.45 |
