# Supplementary material for: Expression Regulation Mechanisms of Sea Urchin (Strongylocentrotus intermedius) Under the High Temperature: New Evidence for the miRNA-mRNA Interaction Involvement
Source: Front Genet. 2022 Jun 29;13:876308. doi: 10.3389/fgene.2022.876308 (PMC9277089; doi:10.3389/fgene.2022.876308)
Supplement: Supplementary file 8 [file Table3.DOCX]

| Sample | Valid reads | Mapped reads | Unique Mapped reads | Multi Mapped reads | PE Mapped reads | Reads map to “+” | Reads map to “-” | Non-splice reads | Splice reads |
| --- | --- | --- | --- | --- | --- | --- | --- | --- | --- |
| NR1 | 49711912 | 22761462(45.79%) | 14683122(29.54%) | 8078340(16.25%) | 18361280(36.94%) | 10651545(21.43%) | 10647942(21.42%) | 14201230(28.57%) | 7098257(14.28%) |
| NR2 | 37812016 | 24750429(65.46%) | 16008890(42.34%) | 8741539(23.12%) | 20847138(55.13%) | 11567972(30.59%) | 11596735(30.67%) | 15942706(42.16%) | 7222001(19.10%) |
| NR3 | 46463046 | 32617681(70.20%) | 20524599(44.17%) | 12093082(26.03%) | 27996376(60.26%) | 15030037(32.35%) | 15041868(32.37%) | 18172471(39.11%) | 11899434(25.61%) |
| HR1 | 40255610 | 24777031(61.55%) | 15907411(39.52%) | 8869620(22.03%) | 20868548(51.84%) | 11598149(28.81%) | 11615824(28.86%) | 15981610(39.70%) | 7232363(17.97%) |
| HR2 | 48242788 | 22674858(47.00%) | 14674984(30.42%) | 7999874(16.58%) | 18361230(38.06%) | 10590467(21.95%) | 10564899(21.90%) | 13217451(27.40%) | 7937915(16.45%) |
| HR3 | 45648034 | 25763917(56.44%) | 16080452(35.23%) | 9683465(21.21%) | 21313964(46.69%) | 11867282(26.00%) | 11899952(26.07%) | 13442623(29.45%) | 10324611(22.62%) |
| NW1 | 41149336 | 27274015(66.28%) | 17648014(42.89%) | 9626001(23.39%) | 23116342(56.18%) | 12679488(30.81%) | 12672691(30.80%) | 17040825(41.41%) | 8311354(20.20%) |
| NW2 | 32804248 | 16343337(49.82%) | 10846933(33.07%) | 5496404(16.76%) | 13067886(39.84%) | 7706396(23.49%) | 7720227(23.53%) | 10415279(31.75%) | 5011344(15.28%) |
| NW3 | 43252056 | 28548078(66.00%) | 18273215(42.25%) | 10274863(23.76%) | 24384202(56.38%) | 13248495(30.63%) | 13270851(30.68%) | 16971764(39.24%) | 9547582(22.07%) |
| HW1 | 46498588 | 29024486(62.42%) | 18516226(39.82%) | 10508260(22.60%) | 24637948(52.99%) | 13529827(29.10%) | 13550836(29.14%) | 18245071(39.24%) | 8835592(19.00%) |
| HW2 | 43881072 | 28424596(64.78%) | 18350155(41.82%) | 10074441(22.96%) | 24169366(55.08%) | 13350938(30.43%) | 13359680(30.45%) | 18362589(41.85%) | 8348029(19.02%) |
| HW3 | 48065808 | 30201697(62.83%) | 18876384(39.27%) | 11325313(23.56%) | 25870240(53.82%) | 13969102(29.06%) | 13928925(28.98%) | 16554298(34.44%) | 11343729(23.60%) |

Supplementary Table 3 Mapping rates between reads and the reference genome
