# Supplementary material for: Expression Regulation Mechanisms of Sea Urchin (Strongylocentrotus intermedius) Under the High Temperature: New Evidence for the miRNA-mRNA Interaction Involvement
Source: Front Genet. 2022 Jun 29;13:876308. doi: 10.3389/fgene.2022.876308 (PMC9277089; doi:10.3389/fgene.2022.876308)
Supplement: Supplementary file 9 [file Table4.DOCX]

Supplementary Table 4 Distribution of reference genome mapping regions

| Samples | NR1 | NR2 | NR3 | HR1 | HR2 | HR3 | NW1 | NW2 | NW3 | HW1 | HW2 | HW3 | avarage |
| --- | --- | --- | --- | --- | --- | --- | --- | --- | --- | --- | --- | --- | --- |
| exon | 80.40 | 78.80 | 82.82 | 79.27 | 81.29 | 82.11 | 79.94 | 76.38 | 80.88 | 79.63 | 79.96 | 80.86 | 80.20 |
| intron | 5.77 | 5.44 | 4.96 | 5.28 | 5.26 | 5.13 | 5.05 | 5.38 | 5.41 | 5.12 | 5.05 | 5.20 | 5.25 |
| intergenic | 13.83 | 15.77 | 12.22 | 15.45 | 13.45 | 12.75 | 15.01 | 18.24 | 13.71 | 15.25 | 14.99 | 13.94 | 14.55 |
